# Light‐Operated Transient Unilateral Adhesive Hydrogel for Comprehensive Prevention of Postoperative Adhesions

**DOI:** 10.1002/advs.202403626

**Published:** 2024-06-25

**Authors:** Furong Cui, Shihong Shen, Xiaoxuan Ma, Daidi Fan

**Affiliations:** ^1^ Engineering Research Center of Western Resource Innovation Medicine Green Manufacturing Ministry of Education School of Chemical Engineering Northwest University Xi'an 710069 China; ^2^ Shaanxi Key Laboratory of Degradable Biomedical Materials and Shaanxi R&D Center of Biomaterials and Fermentation Engineering School of Chemical Engineering Northwest University Xi'an 710069 China; ^3^ Biotech. & Biomed. Research Institute Northwest University Xi'an 710069 China

**Keywords:** anti‐postoperative adhesion, hydrogen bond‐modulated hydrogel, light‐operated, unilateral tissue adhesion

## Abstract

Dislocation of anti‐adhesion materials, non‐specific tissue adhesion, and the induction of secondary fibrinolysis disorders are the main challenges faced by postoperative anti‐adhesion materials. Herein, a self‐leveling transient unilateral adhesive hydrogel is custom‐designed to conquer these challenges with a theoretically calculated and dual‐step tailored gellan gum (GG) as the sole agent. First, the maximum gelation temperature of GG is lowered from 42–25 °C through controlled perturbation of intra‐ and inter‐molecular hydrogen bonds, which is achieved by employing the methacrylic anhydride as a “hydrogen bond's perturbator” to form methacrylate GG (MeGG). Second, the “self‐leveling” injectability and wound shape adaptably are endowed by the formation of borate‐diol complexed MeGG (BMeGG). Finally, the transient unilateral tissue‐adhesive hydrogel (BMeGG‐H) barrier is prepared through photo‐controlled cross‐linking of reactive alkenyl groups. This degradable hydrogel demonstrates favorable rheological properties, light‐controlled unilateral adhesion properties, biocompatibility, anti‐fibrin adhesion, and anti‐cell adhesion properties in vitro. Comprehensive regulation of the fibrinolysis balance toward non‐adhesion is conformed in a rat model after intra‐abdominal surgery via anti‐autoinflammatory response, intestinal wall integrity repair, and Tissue plasminogen activator (t‐PA) and plasminogen activator inhibitor‐1 (PAI‐1) balance adjustment. Notably, the 14^th^ day anti‐adhesion effective rate is 100%, indicating its significant potential in clinical applications for postoperative anti‐adhesion.

## Graphical abstract

This is a hydrogen bond‐modulated self‐leveling and transient unilateral adhesive hydrogel for comprehensive prevention of postoperative adhesions. This hydrogel overcomes previous challenges of anti‐postoperative adhesion materials including dislocation of anti‐adhesion materials, non‐specific tissue adhesion, and the induction of secondary fibrinolysis disorders, implementing a comprehensive anti‐adhesion treatment strategy involving immune modulation, fibrinolysis balance regulation, and intestinal wall integrity repair.

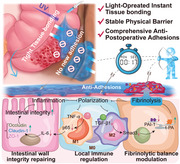

## 1 Introduction

Postoperative adhesion is a common complication of abdominal surgery, occurring in up to 93% of cases despite the widespread adoption of laparoscopic techniques.^[^ ^1^ ^]^ These abdominal adhesions arise due to the abnormal tissue remodeling processes triggered by injury, but their precise mechanisms remain poorly understood.^[^ ^2^ ^]^ The direct causes of postoperative adhesion commonly include imbalances in the fibrinolysis system,^[^ ^3^ ^]^ compromised intestinal wall integrity,^[^ ^4^ ^]^ the infiltration of inflammatory cells, and the excessive release of inflammatory factors,^[^ ^5^ ^]^ all of which result in abnormal extracellular matrix (ECM) deposition.^[^ ^6^ ^]^ Drug‐based prophylaxis and the use of physical barriers are proactive measures for preventing intra‐abdominal adhesion.^[^ ^7^ ^]^ Nonetheless, drug prophylaxis is limited by insufficient spatiotemporal targeting and rapid clearance by the reticuloendothelial system, and drug‐based anti‐adhesion solutions have a failure rate as high as 85%.^[^ ^8^ ^]^ Thus, physical barriers have emerged as the most straightforward approach for adhesion prevention.^[^ ^9^ ^]^ However, most existing physical barrier materials lack self‐adhesive properties, necessitating the use of surgical sutures.^[^ ^10^ ^]^ This causes secondary injuries, enables limited wound coverage, and heightens the risk of barrier displacement.^[^ ^11^ ^]^ Furthermore, it is incompatible with the requirements of minimally invasive surgery.^[^ ^12^ ^]^ Therefore, the clinical efficacy of current anti‐adhesive strategies is significantly short of expectations.^[^ ^13^ ^]^ To this end, the development of injectable anti‐adhesion barrier materials with exceptional wound coverage capabilities, rapid unilateral tissue‐adhesive properties, and robust biological activity is paramount.

Biodegradable hydrogel with wet self‐adhesiveness is considered an ideal candidate for abdominal anti‐adhesion barriers.^[^ ^14^ ^]^ However, most existing self‐adhesive anti‐abdominal adhesion hydrogel patches lack specificity to wound tissues.^[^ ^15^ ^]^ They exhibit indiscriminate tissue adhesiveness, reducing the gap between wound surfaces and altering the relative positioning of open incisions, ultimately exacerbating postoperative adhesion.^[^ ^16^ ^]^ The development of asymmetric adhesive hydrogels is a feasible exploration to solve this problem and holds practical significance. Previously, Cui et al. prepared a unilateral adhesive hydrogel by modifying the surface of carboxyl‐functionalized hydrogels with cationic oligosaccharides via electrostatic complexation after gelation.^[^ ^17^ ^]^ However, their post‐modified anti‐adhesive hydrogel patches could not be injected and failed to adapt to complex wound shapes, thereby increasing surgical complexity and the risk of adhesion. Given that an injectable amphiphilic ionic hydrogel with in situ click gelation performance could solve this issue, Guo et al. prepared an injectable protein‐resistant hydrogel from starch‐graft‐poly (sulfobetaine methacrylate), demonstrating the convenience and effectiveness of injectable amphiphilic ionizable hydrogels in preventing adhesion.^[^ ^18^ ^]^ Nevertheless, graft modification with amphiphilic ionic groups often extends the degradation time of hydrogels, increasing the risk of prolonged retention in vivo.^[^ ^19^ ^]^ Moreover, amphiphilic ion‐based anti‐adhesion materials often fail to exhibit tissue self‐adhesive properties due to due to interface hindrance from the indiscriminate surface hydration layer.^[^ ^20^ ^]^ Therefore, developing a degradable, injectable hydrogel that self‐levels and offers controlled one‐sided adhesiveness represents a significant and challenging endeavor.

In addition to the operational prerequisites of self‐leveling and asymmetric adhesion, an ideal anti‐adhesion hydrogel should also possess comprehensive fibrinolysis balance‐regulating activities, including mitigating autoinflammatory responses, facilitating intestinal wall integrity repair, and adjusting the t‐PA/PAI‐1 balance.^[^ ^21^ ^]^ GG is a highly biocompatible polyanionic natural polymer polysaccharide.^[^ ^22^ ^]^ Polyanionic ligands can regulate the t‐PA/PAI‐1 balance and inhibit tissue inflammation by acting as a physical barrier and polyanion trap to neutralize scavenger receptors.^[^ ^23^ ^]^ Furthermore, GG possesses an inherent ability to resist non‐specific protein adsorption, making it an ideal backbone molecule for anti‐adhesion hydrogels.^[^ ^24^ ^]^ However, the application of GG in anti‐adhesion hydrogels is extremely rare due to its maximum gelation temperature is over 42 °C under natural conditions. This high maximum gelation temperature arises from robust intra‐ and inter‐molecular hydrogen bonding of GG molecules, rendering any operation based on GG molecules impractical under both room temperature and body temperature conditions.^[^ ^25^ ^]^ Drawing inspiration from the research on the role of intramolecular and intermolecular hydrogen bonds in the regulation of silk protein gel properties by Jiang, Wang et al., we believe that employing a strategy involving the controlled disruption of hydrogen bonds by employing a “hydrogen bond's perturbator” holds promise for breaking the limitations of GG applications in vivo.^[^ ^26^ ^]^ Moreover, dynamic cross‐linking based solely on hydrogen bonds results in low bond strength, high‐temperature sensitivity, and unstable tissue adhesion for GG.^[^ ^27^ ^]^ Therefore, covalent cross‐linking is required to strengthen the network for in vivo applications. The employing of the cross‐linkable “hydrogen bond's perturbator” is expected to introduce an additional conditionally triggered covalent cross‐linking network. Considering that reactive alkenyl groups and thiol groups are widely present in proteins, polysaccharides, and lipids within tissues,^[^ ^28^ ^]^ the use of methacrylic anhydride (MA) as the photo‐triggered “hydrogen bond perturbator” could endow a hydrogel with transient asymmetric tissue adhesiveness.^[^ ^29^ ^]^ Additionally, the introduction of relatively inert dynamic boronate ester bonds can endow hydrogel with “self‐leveling” properties before the permanent covalent photo‐crosslinking.^[^ ^30^ ^]^ This shape adaptability renders hydrogel patch to irregularly shaped wounds and tiny perforations on abdominal and intestinal walls, thus reinforcing cell connections in them, and consequently repairing intestinal wall integrity and promoting intestinal wound healing. Ultimately, these comprehensive designs are expected to shift the fibrinolysis balance toward anti‐adhesion by inhibiting TNF‐α, TGF‐β1, and IL‐6 factor‐mediated inflammatory responses.^[^ ^31^ ^]^

**Scheme 1:**
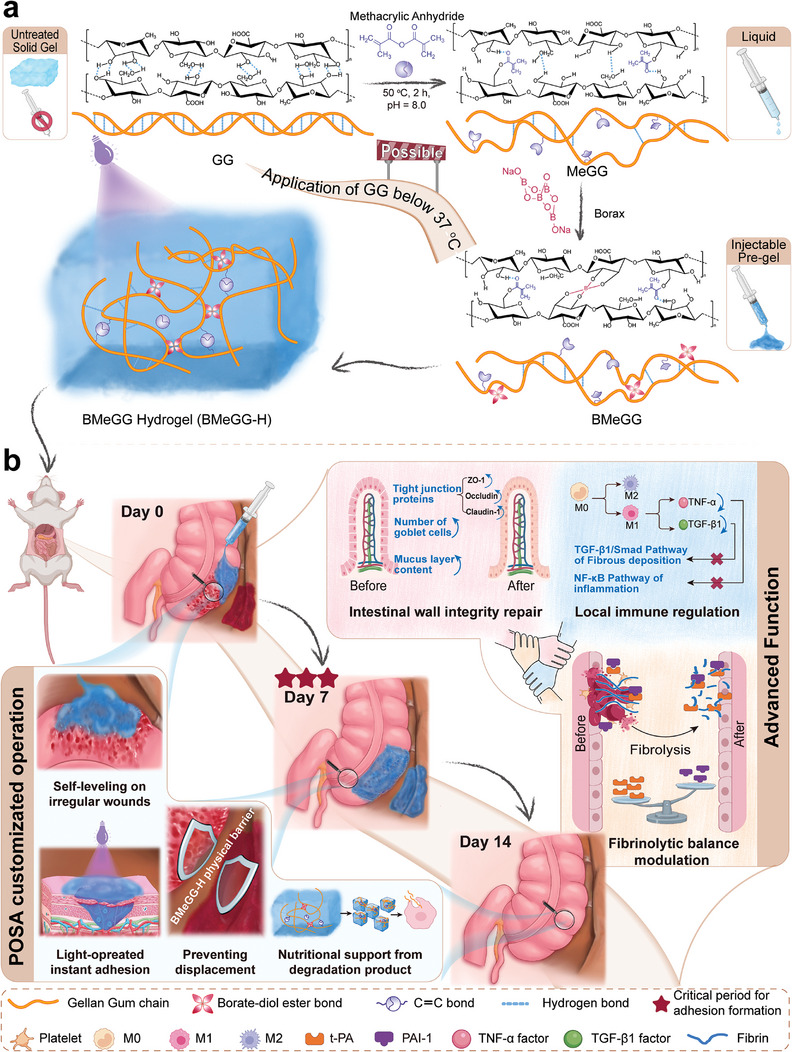
a) Synthesis of the BMeGG‐H hydrogel and b) its therapeutic anti‐adhesion effects in a rat cecum‐abdominal model following surgery.

In this study, a novel hydrogen bond‐modulated self‐leveling and transient unilateral tissue‐adhesive GG hydrogel for the prevention of postoperative adhesion was designed and fabricated. The primary causes underly anti‐adhesion failure with current anti‐adhesion materials were taken into consideration and focused on in this hydrogel patch, including single anti‐adhesion mechanism, limited wound coverage, increased risk of displacement during movement, and non‐wound‐specific adhesion issues. A dual‐step tailored anionic polysaccharide GG was chosen as the sole scaffold molecule and therapeutic agent in this system (**Scheme** **1**). The operational temperature‐related constraints of GG‐based hydrogels for in vivo application were eliminated by employing the methacrylic anhydride as the “hydrogen bond's perturbator” and the formation of the methacrylated GG (MeGG). The irregular and uneven wounds adaptive “self‐leveling” rheological property was endowed by the formation of borate‐diol complexed MeGG (BMeGG). Transient unilateral tissue adhesion was accomplished through light‐operated radical crosslinking of active carbon‐carbon double bonds between the interface of tissue and hydrogel. The biocompatibility, in vitro anti‐fibrin adhesion, and anti‐cell adhesion properties, as well as the comprehensive regulation of the fibrinolysis balance serving for non‐adhesion in vivo, were verified on a standardized rat model following intra‐abdominal surgery. This is a cost‐effective GG‐based hydrogel system with favorable postoperative anti‐adhesion rate, which will provide a prospective insight for the development of novel anti‐adhesive materials.

## 2 Results and Discussion

### 2.1 Synthesis and Characterization of BMeGG‐H

#### 2.1.1 Temperature Constraints Broken of GG and Synthesis of BMeGG‐H

MeGG is synthesized by grafting varying amounts of MA (methacrylic anhydride) onto the β‐D‐glucose of GG and subsequently complexed with borax to form BMeGG (Scheme 1; Table S1, Supporting Information). The details of the synthesis process are presented in Sections 1.4 and 1.5 of the Supporting Information. BMeGG‐H was fabricated via light (365 nm)‐induced crosslinking under 2‐Hydroxy‐4′‐(2‐hydroxyethoxy)−2‐methylacetophenone (I2959) catalysis. At a light irradiation density of 7.14 mW cm^−^^2^, the gelation time of BMeGG‐H was as short as 17 ± 2 s. UV irradiation at this intensity and duration had no significant effect on cell viability and growth (Figure S1, Supporting Information).

**Figure 1:**
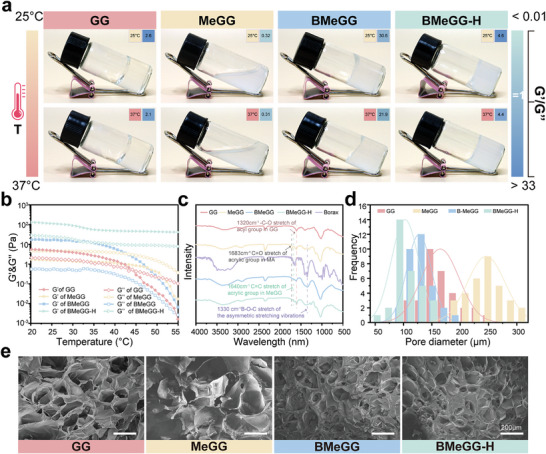
Synthesis and characterization BMeGG‐H. a) The macroscopic photographs of GG, MeGG, BMeGG, and BmeGG‐H at different temperatures. b) The rheological property changes of various hydrogels with temperature. c) FT‐IR spectra of each sample. d) SEM observation and e) Pore diameter analysis of the characterization of GG, MeGG, BMeGG, and BMeGG‐H (scale bars = 200 µm).

A previous study demonstrated that under normal conditions, the maximum gelation temperature of GG is over 42 °C.^[^ ^26^ ^]^ This high maximum gelation temperature is attributed to robust intra‐and inter‐molecular hydrogen bonding of GG molecules, which limits the usability of GG‐based hydrogels at room temperature and their in vivo application (body temperature).^[^ ^31^ ^]^ The phase state of the samples at different temperatures was observed intuitively through an inversion test and qualitatively analyzed using G’/G″ measurements (G’/G″> 1: solid, G’/G″ = 1: viscoelastic, G’/G″< 1: liquid) (**Figure** **1**a). The results showed that 2% GG forms a gel at temperatures between 25 and 37 °C, while MeGG remains in a liquid state within this temperature range. The values of G’/G″ confirmed their solid and liquid properties, respectively (Figure 1b). These findings suggested that MA grafting expanded the temperature application range of GG. Both BMeGG and BMeGG‐H remained in the gel state within this temperature range. However, BMeGG‐H showed a higher G’/G″ than BMeGG, which was indicative of its enhanced solidification properties due to stronger cross‐linking forces.

#### 2.1.2 Chemical Structure of BMeGG‐H

NMR‐H1 and FT‐IR were used to characterize the chemical structure of BMeGG‐H. The degree grafting (GD) of MA on GG was calculated as 7.39–25.33% in different samples, based on Figure S2 and Equation S1 (Supporting Information). FT‐IR findings further confirmed the composition of BMeGG‐H and bond alterations during the synthesis process. After MA grafting, the samples showed the characteristic absorption peak of the carbon double bond at 1640 cm^−1^ and the characteristic C═O peak of ester bonds ≈1770–1680 cm^−1^.^[^ ^32^ ^]^ Upon the addition of borax, BMeGG and BMeGG‐H both exhibited characteristic absorption peaks at 1330 and 1420 cm^−1^ deriving from asymmetric stretching vibration of the B─O─C group.^[^ ^33^ ^]^ After photopolymerization, BMeGG‐H exhibited a new absorption peak at 1640 cm^−1^, which was attributed to the stretching vibration of newly formed acrylic groups post‐photopolymerization (Figure 1c).^[^ ^32^ ^]^ Scanning electron microscopy (SEM) and pore size analysis of freeze‐dried samples with equal solid content revealed a sequentially densifying cross‐linked network and gradually reducing pore size distribution in MeGG, GG, BMeGG, and BMeGG‐H (Figure 1d,e). This trend was consistent with the numerical variations in G’/G″ and highlighted the impact of each synthesis step on the intra‐and inter‐molecular cross‐linking within the final hydrogel system. All these results confirmed the successful formation of the BMeGG‐H, and indicated that the BMeGG‐H was composed of GG grafted from MA, complexed with borax, and crosslinked by Crosslinked via borate ester bonds and active carbon‐carbon double bonds.

### 2.2 Hydrogen Bond‐Modulated Self‐Leveling Capacity

**Figure 2:**
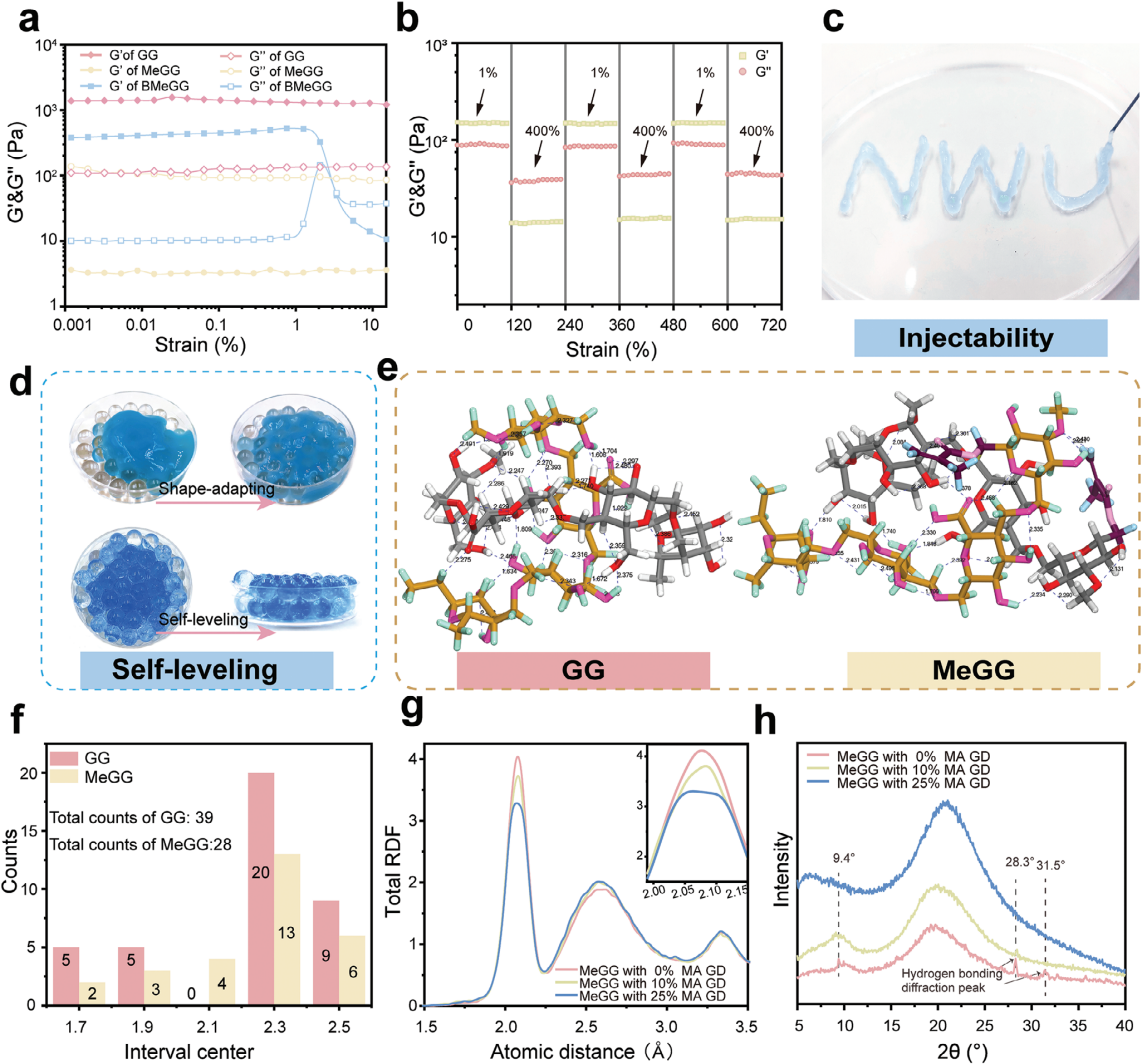
Hydrogen bond‐modulated self‐leveling capacity of BMeGG. a) G′ and G′′ of the BMeGG hydrogel (before light‐induced crosslinking) with different MA GDs under the strain amplitude sweep test. b) G′ and G′′ of the BMeGG hydrogel with an MA GD of 10% under the strain amplitude sweep test. c) Injectable and d) self‐leveling performances of the hydrogel. e) Formation and frequency counts of intra‐ and f) inter‐molecular hydrogen bonds between GG and MeGG monomers. g) Ripley‐dickens function (RDF) analysis of MeGG molecules with MA GDs of 0%, 10%, and 25%. h) XRD spectra of MeGG molecules with MA GDs of 0%, 10%, and 25%.

Wounds in the abdominal wall are typically uneven and irregularly shaped.^[^ ^34^ ^]^ Hence, it is important that anti‐adhesion hydrogels exhibit self‐leveling properties. Shear thinning and dynamic cross‐linking are vital for the injectability and self‐leveling capacity of hydrogels.^[^ ^35^ ^]^ When the strain exceeds the critical point of shear thinning, the crosslinked structure breaks down, causing a transition to the fluid state.^[^ ^36^ ^]^ The injectability and self‐leveling performance of the pre‐crosslinked BMeGG hydrogel (before light‐induced transient unilateral adhesion), regulated by the GD of MA, were evaluated. At 25 °C, the non‐MA‐grafted GG hydrogel did not exhibit shear‐thinning behavior, demonstrating its lack of injectability. BMeGG with a 10% and 25% GD of MA exhibited shear‐thinning characteristics, with the critical shear‐thinning points at 1 rad∙s^−1^ being 3.37% and 112.65% (Figure S3, Supporting Information), respectively (**Figure** **2**a). BMeGG (10% GD of MA) exhibited more suitable mechanical properties and superior tissue retention characteristics. Therefore, the GD of MA was ultimately determined to be 10%. For the borate ester‐based dynamic cross‐linking hydrogel system, the borax content is a key factor, aside from temperature, affecting the self‐leveling properties of the hydrogels. The effect of borate‐diol GD on the injectability and self‐leveling properties of BMeGG was shown in Table S2 (Supporting Information). The optimal borate‐diol GD for the BMeGG system was selected by balancing cytotoxicity with acceptable operation time for abdominal surgery and was ultimately determined to be 1.0%. At this grafting degree, BMeGG could achieved self‐leveling over an area of 4 cm^2^ within 31 ± 4 s. Unless otherwise specified, BMeGG in the following text referred to a 1.0% borate‐diol grafting degree. Alternate strain tests demonstrated that the collapsed structure of the BMeGG hydrogel (10% GD of MA) could immediately recover during cyclic loading and unloading at a high strain of 400% (Figure 2b). This was primarily attributed to the dynamic cross‐linking properties of the borate ester bonds, which ensured the continuity of the crosslinked network in the BMeGG hydrogel after injection and self‐leveling. Macroscopic injectability experiments demonstrated that the BMeGG hydrogel could easily be extruded through a 25 G needle, and it could self‐heal to form the letters “NWU” (Figure 2c). Simulated self‐leveling experiments revealed that the BMeGG hydrogel could fill irregular surfaces and tiny gaps of a circular surface (diameter = 90 mm) within 10 min to generate a smooth self‐leveled interface (Figure 2d).

Since the borate ester bond content remains constant in BMeGG with different GD of MA, the intrinsic intra‐ and inter‐molecular hydrogen bonds in GG appeared to be key regulators of its rheological properties. Molecular dynamics simulations under the framework of the density functional theory (DFT) were carried out to investigate the impact of MA GD on intra‐and intermolecular hydrogen bond formation in MeGG within a water environment. First, stable complex configurations, and hydrogen bond formation in GG and MeGG dual‐monomers were calculated after 1 ns of interaction Figure 2e and Table S3 (Supporting Information). The frequency distribution analysis of intra‐and inter‐molecular hydrogen bond lengths in GG and MeGG indicated that the total number of hydrogen bonds was greater in GG than in MeGG (Figure 2f). Further, the average bond length was shorter in GG. These results suggested that MA effectively perturbs the inherent hydrogen bonds in GG.

The Radial Distribution Function (RDF), a classical molecular dynamics simulation technique was carried out to study poly‐molecular interactions in water.^[^ ^37^ ^]^ With the interaction distance between two atoms as the x‐axis, RDF values represent the probability density of another atom appearing around a central atom, with higher RDF values indicating a more robust interaction between the two atoms.^[^ ^38^ ^]^ RDF computations for GG and MeGG polymer models, each with a polymerization degree of 5 and total chain number of 10, revealed that both GG and MeGG displayed peak RDF values at ≈2.1 Å, consistent with the hydrogen bond distance characteristic of O─H─O interactions (Figure 2g). The order of hydrogen bond peaks in the RDF graph was as follows: 0% GD > 10% GD > 25% GD. Thus, MA grafting perturbed the formation of both intra‐and inter‐molecular hydrogen bonds in MeGG, exerting a stronger hindrance effect on intramolecular hydrogen bonds (Figure S4, Supporting Information). This perturbation was positively correlated with the GD of MA due to spatial steric hindrance.

X‐ray diffraction (XRD) experimental results confirmed the results of the Molecular dynamics simulations. In MeGG, the diffraction peak intensities of intrinsic hydrogen bonds at 28.3° and 31.5°decreased with an increase in the degree of MA grafting. And a similar trend was also observed in the characteristic double‐helix secondary structure diffraction peak at 9.4° (Figure 2h). These findings indicated that MA could act as an effective “hydrogen bond disruptor” in GG‐based systems, thus overcoming temperature application limitations in GG and regulating the self‐leveling properties of BMeGG. This hydrogen bond‐modulated self‐leveling ability could ensure complete coverage of wounds of abdominal/intestinal walls, provide assurance for subsequent light‐operated transient unilateral adhesiveness, and reduce friction and non‐specific postoperative adhesion in the abdominal cavity.

### 2.3 Light‐Operated Transient Unilateral Adhesiveness

**Scheme 2:**
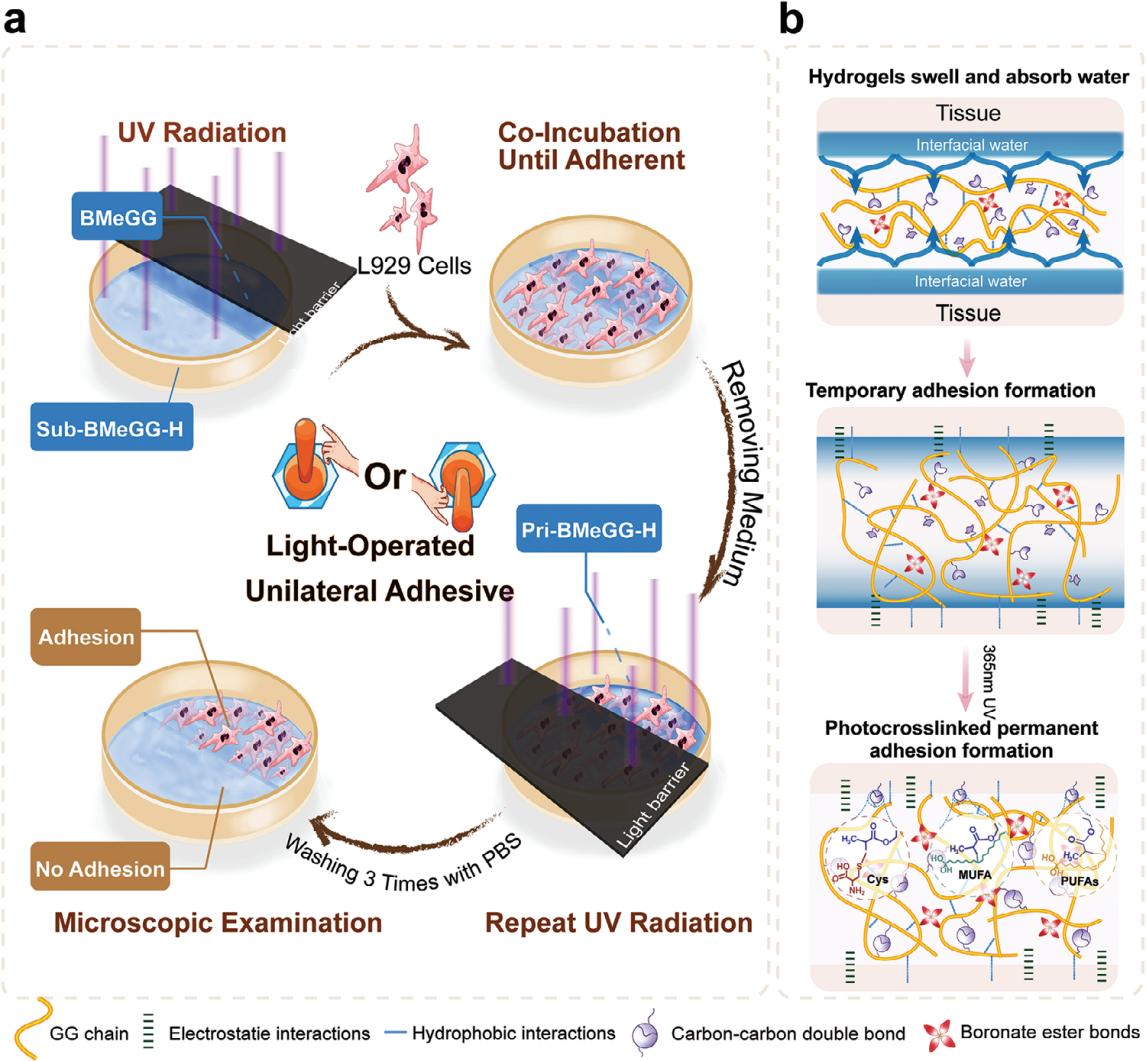
a) Schematic illustration showing the light‐operated transient unilateral adhesiveness of BMeGG‐H to cells and proteins. b) The stepwise mechanism of hydrogel‐tissue interface adhesion.

The primary cause of anti‐adhesion failure within the abdominal cavity is the displacement of physical barriers.^[^ ^39^ ^]^ One effective strategy to tackle this challenge entails the development of hydrogels with unilateral self‐adhesive properties that exclusively adhere to wound tissues without adhering to surrounding tissues.^[^ ^17^ ^]^ Unlike traditional post‐gel surface‐modified hydrogels, BMeGG‐H acquires its unilateral self‐adhesive properties through UV‐initiated radical polymerization involving double bonds on the BMeGG pre‐hydrogel surface and intrinsic double bonds and thiol groups within proteins, polysaccharides, and lipid molecules present within the tissue, such as Cysteine (Cys), Monounsaturated Fatty Acids (MUFA), and Polyunsaturated Fatty Acids (PUFAs). In the presence of light irradiation, BMeGG‐H specifically demonstrated adhesiveness toward damaged tissue. Transient unilateral adhesive was achieved through light‐operated radical cross‐linking of activated carbon‐carbon double bonds and Thiol‐Ene bonds between the tight‐fitting interfaces described above, as shown in **Scheme** **2**b.

**Figure 3:**
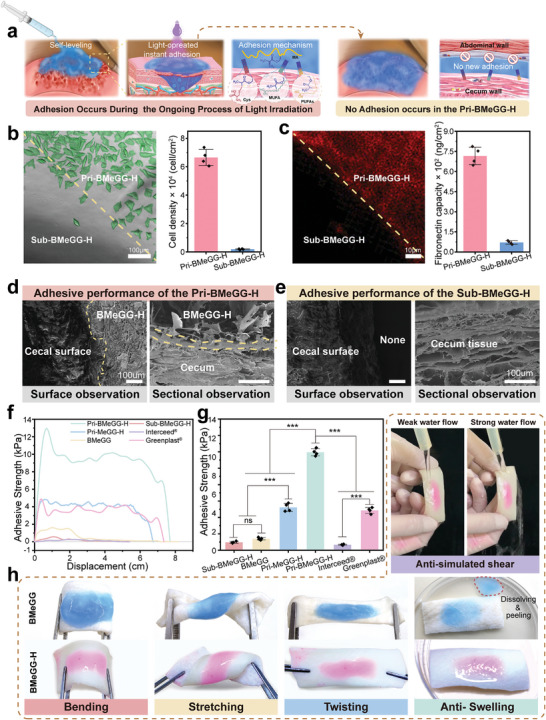
The light‐operated transient unilateral adhesiveness of BMeGG‐H. a) Schematic and mechanism of light‐operated unilateral adhesion. b) Light‐operated transient unilateral adhesion of the BMeGG‐H hydrogel to L929 cells (n = 4) and c) proteins (n = 4). d) Adhesive performance of Pri‐BMeGG‐H (Self‐leveling on the tissue before photo‐crosslinking) and e) Sub‐BMeGG‐H (photo‐crosslinking first before contact with tissue). f) Lap shear tests and g) statistical comparison of the adhesive strength between the hydrogel and appendix tissues (n = 4). h) The shear, tensile, bending, torsional, and swelling resistance properties of BMeGG and BMeGG‐H adhered unilaterally to porcine skin tissue.

Notably, in vivo application of BMeGG typically resulted in the formation of a self‐leveling hydrogel layer with a thickness of 1–2 mm. When the BMeGG hydrogel was 2 mm thick, its transmittance at 365 nm was 74.9% (Figure S5, Supporting Information). In vivo studies demonstrated that this level of transmittance was sufficient to induce photopolymerization at the hydrogel‐tissue interface. Once light irradiation was discontinued, no additional crosslinks could be formed between the tissue and the hydrogel interface. Further, the negatively charged BMeGG‐H hydrogel, rich in hydroxyl groups, prevented protein adsorption through hydrogen bonding and ionic solvation in water, thereby preventing the adhesion of the BMeGG‐H hydrogel to the surrounding tissue (**Figure** **3**a).^[^ ^40^ ^]^ Based on the sequence of photo‐crosslinking and interaction with target agents (proteins, cells, and tissues), BMeGG‐H was termed “Pri‐BMeGG‐H” when contact with the target tissues was established prior to photo‐crosslinking. However, it was termed “Sub‐BMeGG‐H” when contact with the target tissues was established subsequent to photo‐crosslinking. The light‐operated unilateral self‐adhesive properties were investigated at the protein, cellular, and tissue levels.

Type I collagen (Col‐1), known for its high content sites of adhesion, and epithelial cells, which are often in direct contact with anti‐adhesive materials, were employed to evaluate the light‐operated transient unilateral adhesive properties of BMeGG‐H.^[^ ^41^ ^]^ The experimental protocol is shown in Scheme 2. The adhesiveness of the hydrogel to both proteins and cells displayed significant asymmetry across either side of the demarcation line, and the difference was statistically significant (*p* < 0.001). This indicated that the BMeGG‐H hydrogel only adhered to proteins and cells in the presence of light irradiation. Meanwhile, the Sub‐BMeGG‐H hydrogel did not adhere to protein and cells. (Figure 3b,c). SEM was used to image the interfaces after adhesion in order to unveil the microscopic events occurring during the light‐operated adhesion process. Surface observations revealed that Pri‐BMeGG‐H adhered effectively to the surface of the cecal tissue (Figure 3d). Cross‐sectional observations showed that an adhesive layer with a thickness of 5–15 µm was formed between the contact interfaces. This phenomenon was attributed to the outstanding rheological properties of BMeGG, which enabled the subtle infiltration of the tissue. Conversely, no such phenomenon was observed in Sub‐BMeGG‐H (Figure 3e).

The unilateral adhesive strength between the BMeGG‐H hydrogel and the tissue was determined through an overlapping shear experiment.^[^ ^12^ ^]^ A clinically commonly used anti‐adhesive material Interceed and a famous tissue‐adhesive hydrogel Greenplast have been were employed as the control groups.^[^ ^42^ ^]^ As shown in Figure 3f, existing clinical anti‐adhesive materials lack self‐adhesiveness. They are typically sutured onto the damaged peritoneum, inevitably leading to secondary injuries and increased risk of re‐adhesion. Pri‐BMeGG‐H hydrogel exhibits significantly higher adhesive strength than the famous self‐tissue adhesive hydrogel Greenplast (adhesive strength of ≈5 kPa), with multiple independent parallel experiments demonstrating statistically significant differences. The average adhesive strength of Pri‐BMeGG‐H exceeded 11.9 kPa (Figure 3g). Meanwhile, Sub‐BMeGG‐H had no tissue adhesion capacity. The BMeGG pre‐hydrogel possessed a limited tissue adhesion capacity of less than 0.35 kPa and exhibited creep properties. This was due to the formation of hydrogen bonds between the hydroxyl groups in MeGG and the macromolecules within tissues. This temporary adhesion derived from hydrogen bonds endowed the BMeGG pre‐hydrogel with adhesive stability on the tissue before photo‐crosslinking. Pri‐BMeGG‐H showed a better tissue adhesion strength than BMeGG as well as a smaller detachment distance. This indicated that the tissue adhesion capacity of BMeGG‐H was mainly a result of photo‐crosslinking at the adhesive interface, and the extensibility and shear resistance of BMeGG‐H were improved after the incorporation of dynamic borate bonds. Compared with Pri‐MeGG‐H, Pri‐BMeGG‐H showed better extensibility and shear resistance at the adhesive interface owing to the introduction of dynamic borate bonds.

This light‐operated transient unilateral adhesive properties of the hydrogel were also observed on the surfaces of different organs (Figure S6, Supporting Information). Considering the complex forces in the intra‐abdominal environment and the presence of bodily fluids, the shear, tensile, bending, torsional, and swelling resistance properties of BMeGG and BMeGG‐H were observed after unilateral adherence to porcine skin tissue. BMeGG without undergoing photo‐crosslinking reinforcement could self‐level and adhere to the surface of porcine skin tissue, exhibiting significant deformations during shear, tensile, bending, and torsional processes, with poor resistance to swelling in simulated body fluids (Figure 3h). These results underscore the necessity of reinforcing the hydrogel network via UV crosslinking and achieving covalent and permanent hydrogel‐tissue adhesion. The hydrogel remained intact and securely adhered to porcine skin after water flushing (Figure 3h; Movie S1, Supporting Information), stretching, twisting, and immersion in PBS for 24 h. In vitro, swelling experiments indicated that the BMeGG‐H hydrogel had a swelling capacity lower than 73.43% ± 1.23 in PBS, mainly due to the repulsion of water molecules by the grafted hydrophobic MA groups (Figure S7, Supporting Information). These results highlighted the exceptional tissue retention capacity of the BMeGG pre‐hydrogel, the robust adhesive capacities of Pri‐BMeGG‐H on damaged tissue, and the anti‐adhesive properties of Sub‐BMeGG‐H against surrounding tissues. The light‐operated transient unilateral adhesiveness of BMeGG‐H successfully enabled controllable tissue adherence, effectively preventing adhesions and displacement. This property was crucial for the effectiveness of this anti‐adhesion material.

### 2.4 Anti‐Adhesion Efficacy In Vitro

**Figure 4:**
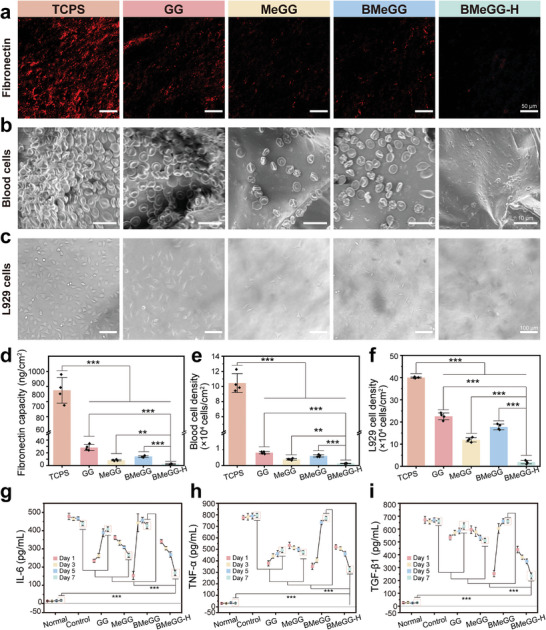
Anti‐adhesion efficacy of BMeGG‐H in vitro. a) Representative fluorescence images of fluorescently labeled fibrinogen adhesion on different hydrogels. b) Representative SEM images of blood cell adhesion on different hydrogels. c) Representative micrographs of L929 cells adhesion on different hydrogels. d) Quantitative analysis of fibrinogen, e) blood cells, and f) L929 cell adhesions, respectively (n = 4). g) The expression of cytokine IL‐6, h) the expression of TNF‐α, and i) the expression of TGF‐β1 over time (n = 4).

The adhesion of fibrinogen and blood cells marks the beginning of adhesion, while the adhesion and aggregation of fibroblasts play a pivotal role in the progression of adhesive lesions.^[^ ^43^ ^]^ The adhesion capabilities of BMeGG‐H to fibrinogen (**Figure** **4**a), blood cells (Figure 4b; Figure S8, Supporting Information), and L929 cells (Figure 4c) after co‐incubation were employed to evaluate its in vitro anti‐adhesion properties. Compared to TCPS (tissue culture plate surface), the four GG‐based hydrogels exhibited resistance to fibrinogen adhesion in varying degrees. Among them, BMeGG‐H demonstrated the strongest protein adhesion resistance (*p* < 0.001). In contrast to the GG group, the MeGG after photo‐crosslinking group showed enhanced anti‐fibrinogen adhesion properties. Surprisingly, BMeGG, with the introduction of borate ester bonds prior to photo‐crosslinking, shows a slight reduction in protein resistance compared to the MeGG group (Figure 4d). The blood cells involved coagulation cascade reaction could induce the excessive deposition of fibrin by activating thrombin, ultimately leading to the formation of adhesions.^[^ ^43^ ^]^ In comparison to TCP group, all GG‐based hydrogel groups exhibited excellent anti‐adhesive properties to blood cells (Figure 4e) and fibroblast L929 cells (Figure 4f). Furthermore, the adhesion amounts decrease in the order of GG, BMeGG, MeGG, and BMeGG‐H, following a similar trend with fibrinogen adhesion.

The inherent anti‐adhesion properties of GG could be attributed to three main factors. First, the negatively charged carboxyl groups on the glucuronic acid residues within the GG molecules could effectively prevent the adsorption of negatively charged proteins and cells. Second, as an anionic polysaccharide, GG could establish a protective hydration layer through hydrogen bonding and ion solvation, thus preventing the induction of adhesions. Third, the secondary structure of GG molecules could consume a significant number of internal hydrogen bonds, thereby reducing the active adhesion groups on the surface. The MeGG hydrogel showed significantly better (*p* < 0.001) anti‐adhesion properties than the GG hydrogel. This was because the photo‐crosslinking process imparted a denser crosslinking structure to the MeGG hydrogel, thereby restricting the activity range of the active adhesion groups on the hydrogel surface. The inherent hydrophobicity of the grafted MA groups in MeGG also contributed to these enhanced anti‐adhesion properties.^[^ ^29^ ^]^ BMeGG produced a slightly weaker anti‐adhesion effect than MeGG. This may be because the stronger rheological properties of BMeGG caused the exposure of active adhesion groups, such as hydrogen bonds, on the hydrogel surface. BMeGG‐H showed the strongest anti‐adhesion effects among all the hydrogels (*p* < 0.001) owing to the inherent anti‐adhesion properties of GG, the grafting of hydrophobic MA, the increased crosslinking density, and a reduction in exposed active adhesion groups due to photo‐crosslinking.

Inflammation‐induced fiber deposition plays a pivotal role in the formation of adhesions, particularly at 5–7 days post‐operation.^[^ ^44^ ^]^ Hence, this period is considered a critical phase for the development of adhesions. TNF‐α, TGF‐β1, and IL‐6 are three key immunoregulatory cytokines secreted by macrophages. IL‐6 can induce adhesions by creating a local inflammatory reaction in wound tissues.^[^ ^31a^ ^]^ TNF‐α can disrupt the fibrinolytic balance by promoting the proliferation of fibroblasts and the synthesis of plasminogen activator inhibitors.^[^ ^45^ ^]^ Meanwhile, TGF‐β1 serves as the primary fibrotic mediator during the formation of adhesions.^[^ ^31a^ ^]^ In this study, hydrogels were co‐cultured with a specific number and density of LPS‐treated macrophages for a fixed duration (1–7 days). Subsequently, the remaining hydrogels were moved to new culture dishes containing the same number and density of macrophage cells, and the concentrations of TNF‐α, IL‐6, and TGF‐β1 in the medium were measured (Figure 4g–i). Compared to the control group, the model group expressed elevated levels of all inflammatory cytokines, indicating the successful establishment of the LPS‐induced in vitro inflammation model. All four GG‐based hydrogels significantly (*p* < 0.001) downregulated the expression levels of TNF‐α, TGF‐β1, and IL‐6 to varying degrees on the first day, attributed to the inherent anti‐inflammatory efficacy of GG (Figure S9, Supporting Information). However, GG exhibited relatively low anti‐inflammatory activity during the observation period due to the slow degradation of its resilient double‐helix structure at 37 °C, resulting in the minimal production of free active degradation products. Meanwhile, although BMeGG displayed strong anti‐inflammatory effects before day 3, its efficacy significantly decreased at 5–7 days. This was due to the absence of covalent cross‐linking in the BMeGG hydrogel, which only contained sparse hydrogen and borate bonds. As a result, the hydrogel network completely disintegrated within 3 days, causing a limited quantity of active substances to remain during the later stages of the experiment. The inhibitory effects of MeGG and BMeGG‐H on the expression of inflammatory factors increased with time. The BMeGG‐H group exhibited the lowest expression of TNF‐α, TGF‐β1, and IL‐6, coinciding with the peak therapeutic window of 5–7 days for anti‐adhesion effects. These in vitro experiments clearly demonstrated that BMeGG‐H exerted a notable inhibitory effect on the pivotal factors responsible for adhesions at the molecular, protein, and cellular levels, which was a prerequisite for anti‐adhesion efficacy in vivo.

### 2.5 Biocompatibility and Host Response

**Figure 5:**
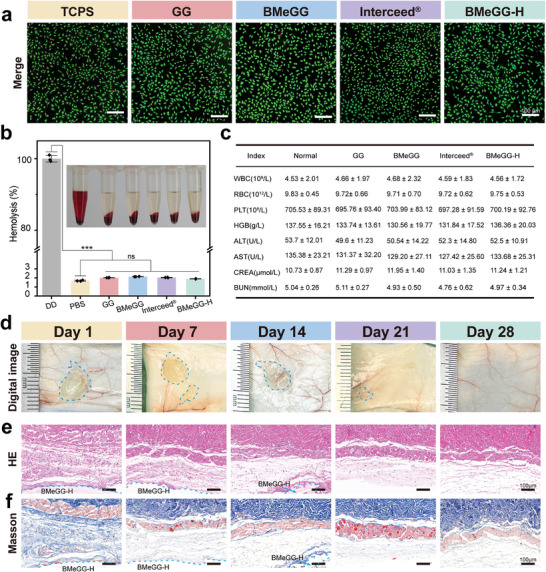
The biocompatibility of BMeGG‐H and the host response. a) Live/Dead staining of mouse fibroblast cells (L929) after co‐incubation with hydrogel samples for 24 h. Scale bar = 100 µm. b) Hemolytic activity of different hydrogels (n = 3). c) Hematological and blood biochemical analyses in BALB/c mice after the subcutaneous implantation of BMeGG‐H. Parameters include the white blood cell count (WBC), red blood cell count (RBC), hemoglobin (HGB), platelet count (PLC), alanine aminotransferase levels (ALT), aspartate aminotransferase levels (AST), creatinine levels (CREA), and blood urea nitrogen levels (BUN) (n = 4). d) Digital photographs, e) H&E staining, and f) Masson's trichrome staining of the BMeGG‐H hydrogel after subcutaneous degradation. The blue dotted area shows the hydrogel remaining after degradation for a certain period.

The cytotoxicity, hemolytic properties, and host responses of the BMeGG‐H hydrogel and its components were evaluated to assess its biocompatibility after subcutaneous implantation.^[^ ^20^ ^]^ Live/dead staining of L929 cells revealed that there was no significant cell death after co‐incubation with various hydrogels for 24 h (**Figure** **5**a). A quantitative Cell Counting Kit‐8 (CCK‐8) assay indicated that the cell viability of all groups was comparable to that of the TCP group (Figure S10, Supporting Information). These results suggested that, under the given experimental conditions, the BMeGG‐H hydrogel and its components did not exhibit apparent cytotoxicity. Hemolysis tests demonstrated the good hemocompatibility of the BMeGG‐H hydrogel (Figure 5b). Evaluation of host responses in mice after subcutaneous implantation of BMeGG‐H hydrogel revealed no significant differences in blood routine, biochemical indicators (Figure 5c), or tissue structure of major organs (heart, liver, spleen, lung, kidney) compared to the blank group (mice without implants) (Figure S11, Supporting Information). All these results indicated that there was no evident in vivo acute or chronic toxicity associated with BMeGG‐H hydrogel implantation.

Furthermore, the in vivo degradation process of the BMeGG‐H hydrogel was digitally recorded and histologically observed. Tissues at the implantation sites were examined using hematoxylin and eosin (H&E) staining and Masson's trichrome staining. The BMeGG‐H hydrogel maintained a stable barrier effect during the critical adhesion period (7–14 days), isolating pre‐adhesion tissues, reducing the occurrence of adhesions, and achieving complete degradation within 28 days (Figure 5d). No signs of congestion, redness, or inflammation were observed at the implantation site throughout the degradation process. Correspondingly, H&E staining of implantation site tissues showed no significant infiltration of inflammatory cells (Figure 5e). Further, Masson's trichrome staining demonstrated that no connective tissue capsule formation had occurred around the implant material and that there was no abnormal collagen deposition after the complete degradation of the BMeGG‐H hydrogel (Figure 5f). Collectively, these findings suggested that BMeGG‐H had excellent biocompatibility, produced desirable host responses after implantation, and exhibited a suitable degradation performance that complemented the anti‐adhesion processes.

### 2.6 Anti‐Adhesion Efficacy In Vivo

**Figure 6:**
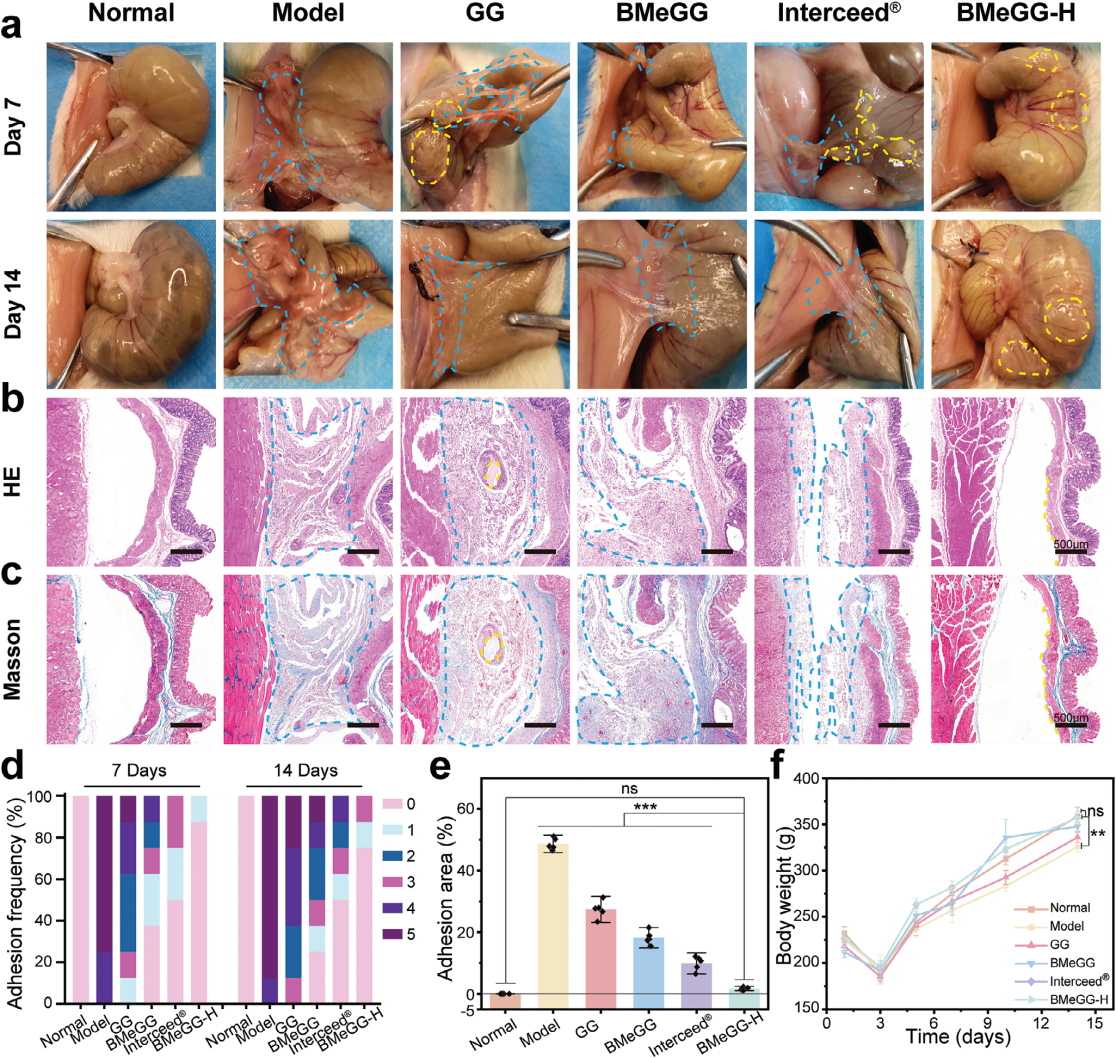
a) Representative photographs depicting the adhesions in each group on postoperative days 7 and 14. b) H&E staining and c) Masson's trichrome staining of typical adhesion tissues on Day 14. d) Adhesion scores on postoperative days 7 and 14 (n = 8). e) Percentage of the adhesion area in each group (with the initial wound area set to 1, n = 8). f) Postoperative changes in the body weight of the rats over time (n = 8). The blue dotted line indicates the adhesion area, and the yellow dotted line indicates the hydrogel remaining after degradation.

The anti‐adhesive efficiency of BMeGG‐H in vivo was evaluated on a rat sidewall defect‐cecum abrasion model, as established in Figure S12 (Supporting Information).^[^ ^46^ ^]^ The in vivo administration process of BMeGG‐H was illustrated in Figure S13 and Movie S2 (Supporting Information). The rats were divided into six groups, as follows: normal group (no abdominal surgery), model group (saline treatment group), GG group, BMeGG group, Interceed group (clinical material), and BMeGG‐H group. Since adhesions typically form 5–7 days after abdominal surgery, and irreversible adhesions tend to form within 7–14 days postoperatively, adhesion status was recorded at both 7 and 14 days after surgery (**Figure** **6**a). Areas of adhesion were delineated with red dashed lines, while the remaining anti‐adhesion materials were marked with blue dashed lines. Adhesion degrees were qualitatively assessed following a standard scoring system (Table S4, Supporting Information).^[^ ^47^ ^]^

H&E staining and Masson's trichrome staining were observed microscopic changes at the sites of adhesion and were conducted to assess the abnormal collagen deposition and injuries of the cecum and abdominal walls at the adhesion sites on the 14^th^ day (Figure 6b,c). On the 14 Days postoperative, the model group, GG group, and BMeGG group exhibited varying degrees of adhesions, along with noticeable abnormal collagen deposition and inflammatory cell infiltration in serosal layers of the cecum and abdominal wall. This phenomenon was not observed in the BMeGG‐H group, indicating that BMeGG‐H exerted in vivo anti‐adhesive effects by suppressing inflammation and promoting wound healing of the intestinal wall and abdominal wall. Observations of the abdominal tissue in the model group revealed the presence of significant adhesions on Day 7 and irreversible deep tissue adhesions on Day 14, with average scores of 4.3 ± 0.2 and 4.5 ± 0.2, respectively. This confirmed the successful establishment of the postoperative adhesion model. The GG group and BMeGG group exhibited moderate amounts of mucosal adhesions, with average scores of 3.3 ± 0.4 and 2.5 ± 0.2, respectively, on Day 7. On Day 14, the adhesions in both groups intensified further, and deep‐seated adhesions were detected in some samples (Figure 6d). The adhesion area was calculated using Image J (Figure 6e). These results indicated that although the GG hydrogel can mitigate postoperative adhesions to some extent given its intrinsic anti‐adhesion properties and its ability to enhance wound healing, it still cannot prevent adhesion development. This was primarily because GG lacks inherent tissue‐adhesive properties, which rendered it incapable of preventing adhesion failure due to displacement.^[^ ^27^ ^]^ Meanwhile, the BMeGG group exhibited better anti‐adhesion effects than the GG group on Day 7. However, this advantage disappeared on Day 14. This was likely because the self‐leveling BMeGG hydrogel provided more robust wound coverage in the early stages of adhesion progression. However, its rapid degradation (< 7 days) due to weak cross‐linking led to the dissolution of the anti‐adhesion physical barrier in the later stages. In the BMeGG‐H group, a light‐operated tightly adherent protective barrier was observed at the site of cecum injury, and the surrounding tissues did not show adhesions. On Day 14, the integrity of both the cecum and abdominal wall was restored, leaving no residual postoperative wounds. The adhesion scores on days 7 and 14 (0.3 ± 0.1 and 0.1 ± 0.1, respectively) were the lowest in the BMeGG‐H group (Table S5; Table S6, Supporting Information). Additionally, BMeGG‐H could undergo complete degradation over ≈14 days in vivo and produce a good host response during degradation. This ensured that BMeGG‐H could maintain a stable physical barrier throughout the duration of adhesion formation, without impeding wound healing (Figure 5).

Furthermore, in contrast to the restricted weight gain observed in other treatment groups, the weight gain curve of the rats in the BMeGG‐H group showed no significant difference with that of the normal group (*p* > 0.05), suggesting a positive correlation between promoting intestinal function recovery in rats and its strong anti‐adhesive efficacy under BMeGG‐H treatment (Figure 6f). These findings demonstrated the in vivo anti‐adhesive effectiveness of BMeGG‐H based on its self‐leveling properties, light‐operated transient unilateral adhesiveness, and high biological activity.^[^ ^48^ ^]^

### 2.7 Analysis of Trinity Anti‐Adhesion Routes

#### 2.7.1 Regulation of the Fibrinolytic Protein Balance by BMeGG‐H

**Figure 7:**
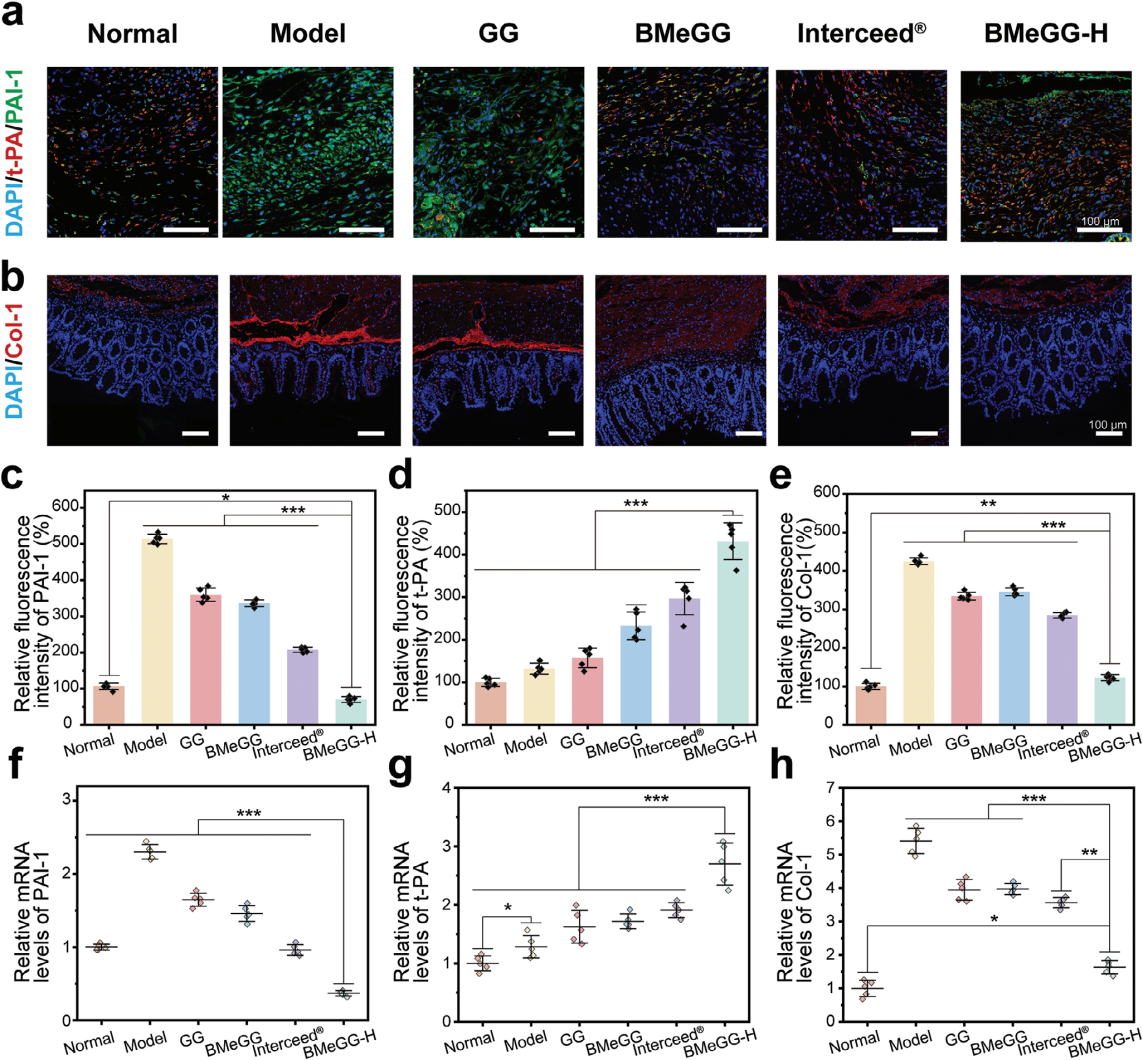
The fibrinolytic protein balance regulation of BMeGG‐H. a) Immunofluorescence staining of DAPI (blue), t‐PA‐AlexaFluor594 (red), and PAI‐1‐AlexaFluor488 (green) in injured tissues on postoperative days 7. b) Immunofluorescence staining of DAPI (blue) and Col‐1 (red) in injured tissues on postoperative days 7. c) Relative fluorescence intensities of PAI‐1, d) t‐PA, and e) Col‐1 mRNA in injured tissues (with the initial wound area set to 1, n = 6). f) Relative mRNA levels of PAI‐1, g) t‐PA, and h) Col‐1 mRNA in injured tissues (with the initial wound area set to 1, n = 6). scale bars = 100 µm.

The cytokines t‐PA and PAI‐1 play crucial roles in regulating fibrinolysis in vivo. Disruption of fibrinolysis balance can lead to abnormal collagen deposition, primarily Col‐1, which in turn promotes the formation of adhesions.^[^ ^49^ ^]^ In this study, the anti‐adhesion ability of BMeGG‐H hydrogel deriving from the regulation of fibrinolytic balance was investigated qualitative and quantitative analysis at protein and gene levels. Immunofluorescence staining for PAI‐1, t‐PA, and Col‐1 in injured tissues was performed on postoperative days 7 and 14 (**Figure** **7**a; Figure S14, Supporting Information). Postoperative tissues in the model group exhibited elevated expression of PAI‐1 (green fluorescence) and reduced expression of t‐PA (red fluorescence) compared to normal tissues.^[^ ^50^ ^]^ The overexpression of PAI‐1 further inhibited the activity of t‐PA, leading to postoperative adhesions. The significant reduction of PAI‐1 expression was not observed following treatment with GG hydrogel, underscoring its limited efficacy in preventing translocation. The overexpressed PAI‐1 was also not attenuated in the BMeGG group that was easily diluted and dissolved. In contrast, in the BMeGG‐H hydrogel group, more red fluorescence (t‐PA) and less green fluorescence (PAI‐1) are observed indicating the significantly promoting and reducing abilities of BMeGG‐H hydrogel on t‐PA and PAI‐1, respectively (Figure 7c,d; Figure S15, Supporting Information). Immunofluorescence staining Col‐1 confirmed the outcomes of fibrinolysis protein balance regulation, underscoring its superior effectiveness in postoperative anti‐adhesion treatment (Figure 7b,e; Figure S16, Supporting Information).

Quantitative RT‐qPCR and ELISA analyses were conducted to assess the protein expression and mRNA transcription levels of PAI‐1 and t‐PA in the injured tissues on Day 7, a critical time point. The mRNA transcription levels of PAI‐1 in panels GG, BMeGG, Interceed, and BMeGG‐H exhibited a sequential decrease (Figure 7f), while t‐PA transcription levels showed the opposite trend (Figure 7g). ELISA demonstrated a similar trend between protein expression and mRNA transcription levels across all groups. Remarkably, PAI‐1 mRNA levels were the lowest while t‐PA expression levels were the highest in the BMeGG‐H hydrogel group, consistent with the trends observed in PAI‐1 and t‐PA protein expression (*p* < 0.001). These quantitative findings confirmed the effectiveness of the BMeGG‐H hydrogel in preventing abnormal collagen deposition (Figure 7h) by shifting the fibrinolytic balance toward fibrinolysis, thus exerting anti‐adhesion effects.

#### 2.7.2 The Intestinal Wall Integrity Reparation of BMeGG‐H

**Figure 8:**
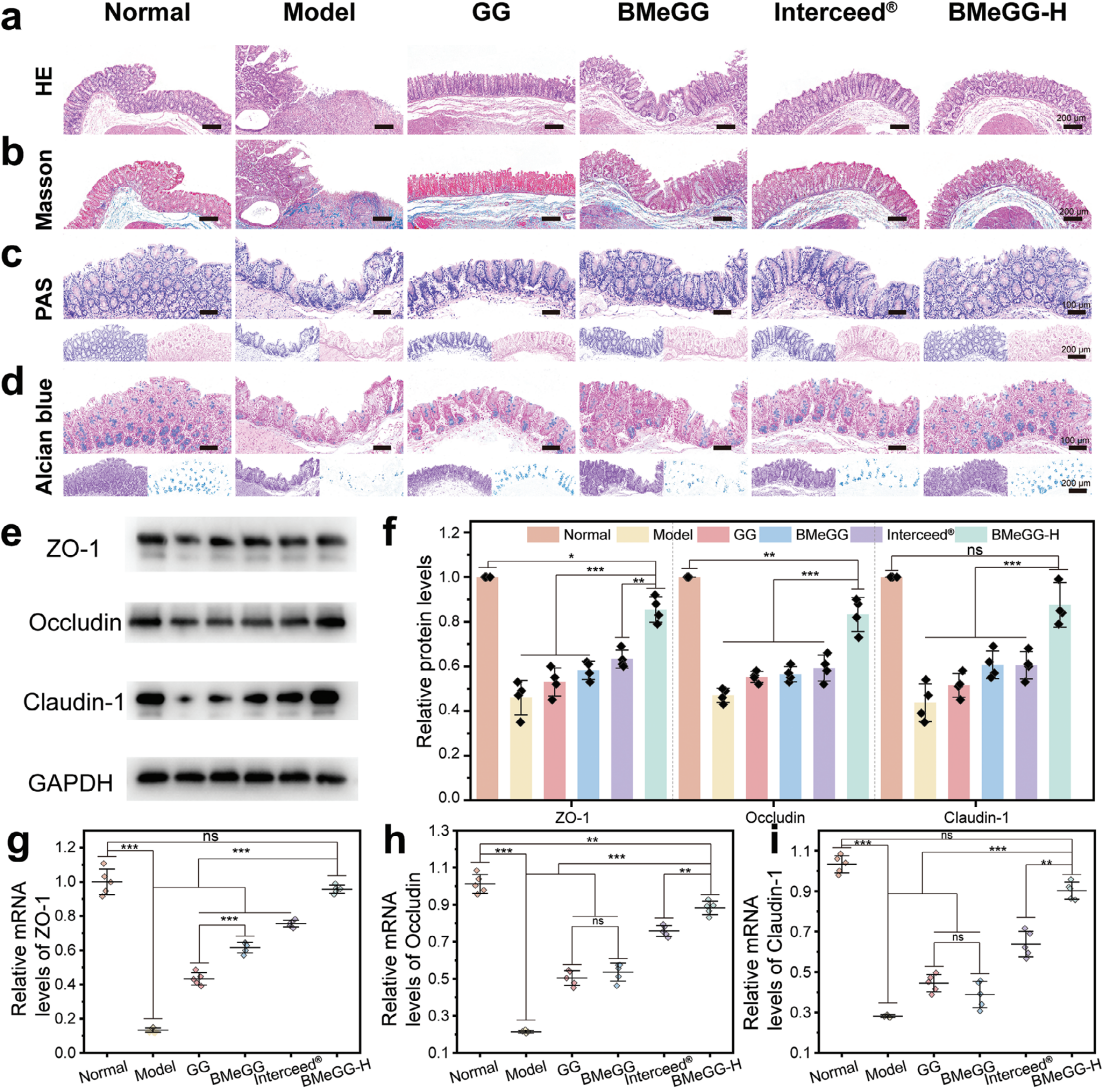
The intestinal wall integrity reparation of BMeGG‐H on the postoperative Day 7. a) Representative images of injured rat cecum tissues stained with H&E and b) Masson's trichrome. c) Representative images of goblet cells stained with Periodic Acid‐Schiff stain (PAS). d) Representative images of mucus layer stained with Alcian blue. e) Western blot detection (n = 4) and f) quantification of tight junction proteins including ZO‐1, Occludin, and Claudin‐1. g) mRNA transcription levels of tight junction proteins (n = 5). Scale bars = 200 µm in (a) and (b), and 100 µm in (c) and (d).

The disruption of intestinal integrity following abdominal surgery is a critical factor exacerbating postoperative intestinal dysfunction and inducing adhesions.^[^ ^51^ ^]^ Significant signs of mucosal ulceration, extensive infiltration of inflammatory cells into the submucosal layer, loss of crypts, and collagen deposition were observed in the damaged cecum mucosa of the model group, compared to healthy cecum tissues.^[^ ^16^ ^]^ Conversely, after the implantation of the BMeGG‐H, there was a notable increase in goblet cells between the injured cecal mucosal epithelial cells and intestinal gland epithelial cells (**Figure** **8**a), and collagen deposition decreased (Figure 8b; Figure S17, Supporting Information). These findings indicated that BMeGG‐H could repair intestinal wall integrity. Furthermore, the mucus produced by goblet cells serves to isolate the contact between intestinal microbiota and host cells, effectively preventing excessive intestinal inflammatory responses, thereby reducing the inflammatory load of the damaged tissue microenvironment and exerting a synergistic anti‐adhesion effect.^[^ ^52^ ^]^ As shown in Figure 8c, the loss of goblet cells and mucus was visibly evident through PAS staining in the model group (Figure S18, Supporting Information), while the loss of goblet cells decreased in all the treatment groups (Figure S19, Supporting Information). Especially In the BMeGG‐H group, goblet cells were well‐preserved both in quantity and structure (Figure 8d). This might be due to the anti‐inflammatory efficacy of BMeGG‐H barrier degradation products, which could effectively anti‐inflammation in the injured cecum area, further preventing epithelial barrier disruption and inhibiting inflammation‐related fiber deposition. Compared to the GG, BMeGG and intercede groups, BMeGG‐H exhibits a greater capacity for restoring intestinal wall integrity and rejuvenating goblet cell function. This is attributed to its tissue retention capabilities conferred by self‐leveling and unilateral instant adhesion, along with its degradation performance corresponding to the wound healing process.

Tight junction proteins, such as Claudin‐1, ZO‐1, and Occludin, play crucial roles in maintaining intestinal function.^[^ ^53^ ^]^ Specifically, claudin‐1 is among the pivotal proteins responsible for upholding the connections between epithelial cells, ZO‐1 is instrumental in maintaining tight junctions between epithelial and endothelial cells, and Occludin is the main backbone of the intercellular connection skeleton. These proteins can regulate intercellular gap permeability and control substance penetration by preserving the stability of cell‐to‐cell connections, thus ensuring the integrity and function of the intestinal tract.^[^ ^54^ ^]^ The expression of tight junction proteins in each group was assessed at the protein level via Western blotting (Figure 8e,f) and mRNA transcription level via q‐PCR (Figure 8g–i). The results showed that all physical barrier treatments were able to upregulate the expression of the three tight junction proteins to some extent. Regrettably, due to differences in their in situ retention capacity, the GG and BMeGG groups failed to achieve the optimal effects. It was worth to note that there was no statistically significant difference in the expression levels of Occludin mRNA and Claudin‐1 mRNA between the BMeGG and GG groups. However, there was a statistically significant difference in ZO‐1 mRNA between the BMeGG and GG groups. This was because after in vivo application, GG rapidly loses its barrier function due to displacement, and BMeGG undergoes rapid in vivo swelling and dilution in body fluids, thus losing its barrier function in the short term too. Therefore, its regulation of tight junctions in appendicitis and the resulting tight junctions in appendix cells mediated by adhesion was limited. The higher expression of ZO‐1 in BMeGG compared to GG might be because ZO‐1 is more sensitive to inflammation compared to the other two tight junction proteins.^[^ ^55^ ^]^ Therefore, the small amount of short‐term staining BMeGG molecules adhered to the surface of damaged appendiceal tissues plays a more significant role in inflammation control based on the “anion trap” mechanism, especially in the early stages of adhesion, affecting ZO‐1. Consistent with the result of the anti‐adhesive effect in vivo, BMeGG‐H had the most pronounced upregulation in the expression of tight junction proteins Claudin‐1, ZO‐1, and Occludin in injured cecal tissues. It could be inferred that part of the protective actions of the BMeGG‐H anti‐adhesion barrier were to mechanically cover the injured cecum and light‐operating bond with it, achieving mechanical pulling and reinforcement of the injured cecum, thereby promoting tissue healing. Additionally, the upregulation of tight junction protein expression helped restore intestinal structure and integrity, maintaining the normal physical barrier function of the intestine and therefore effectively blocking the inflammation‐related fiber deposition process by reducing local inflammation.^[^ ^56^ ^]^

#### 2.7.3 The Immunomodulatory Effect of BMeGG‐H

**Figure 9:**
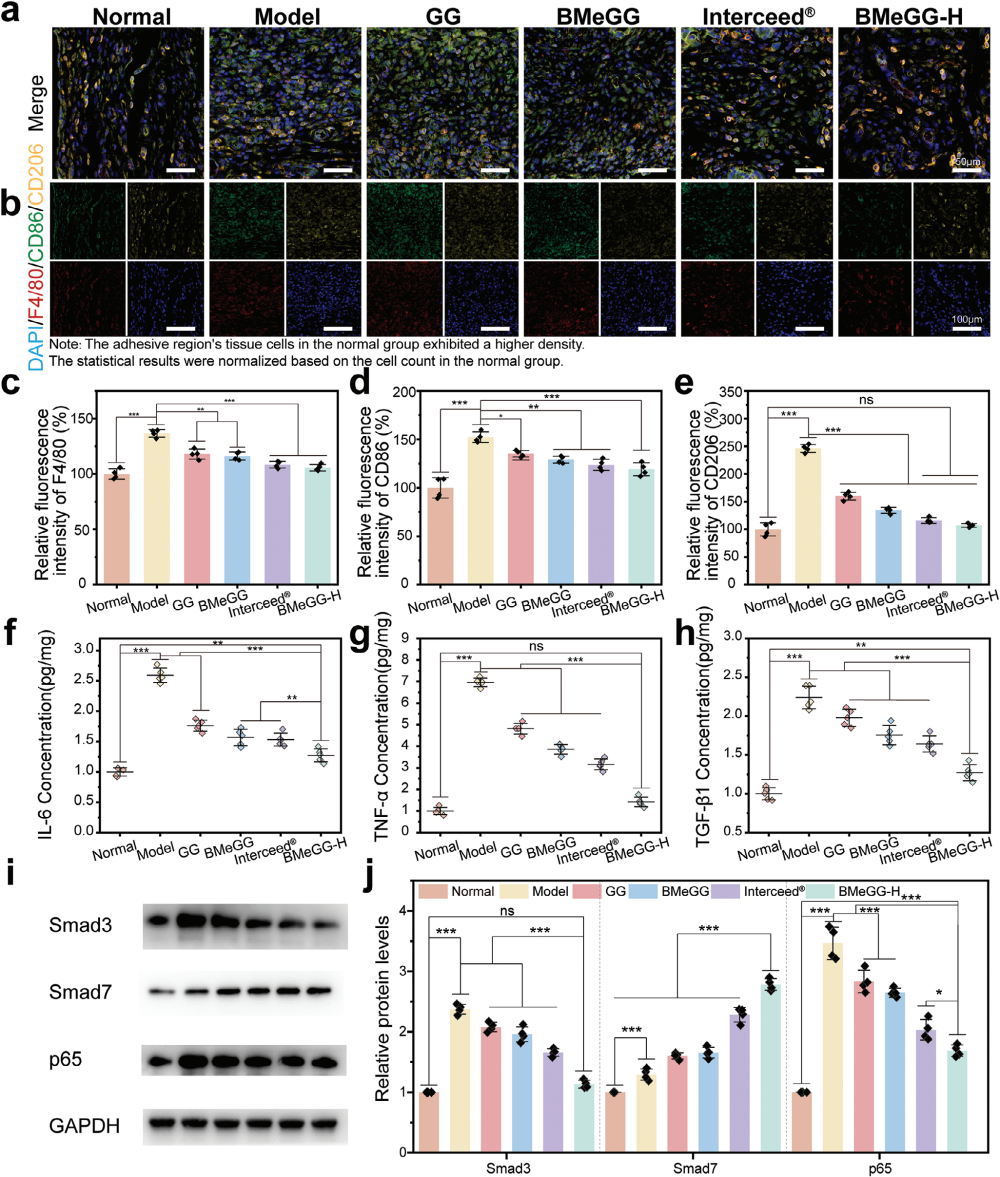
Immunomodulatory effect of BMeGG‐H on postoperative Day 7. a) Immunofluorescence staining merge image of injured rat cecum tissue. b) Immunofluorescence staining of DAPI (blue), F4/80^+^ (red), CD86^+^ (green) and CD206^+^ (yellow) of injured rat cecum tissues. c) Total macrophages. d,e) Relative quantitative analysis of M1 and M2 macrophages (n = 4). f) IL‐6, g) TNF‐α, and h) TGF‐β1 levels in peripheral blood (n = 5). i) Protein expression of p65, Smad3, and Smad7 in injured cecum tissues measured using Western Blot. j) The relative expression of each protein, normalized based on the glyceraldehyde 3‐phosphate dehydrogenase (GAPDH) band (n = 4).

After abdominal and intestinal wall damage, macrophages are activated. These macrophages differentiate into M1‐type macrophages, which can induce inflammation, and M2‐type macrophages, which can promote fiber deposition.^[^ ^57^ ^]^ In this study, immunofluorescence analyses were conducted to assess macrophage behavior on postoperative Day 7, which corresponds to a critical period for adhesion development. First, the total number of macrophages (F4/80^+^), M1 (CD86^+^), and M2 (CD206^+^) macrophages were quantified through immunofluorescence staining (**Figure** **9**a,b). The total number of macrophages (F4/80^+^) in the GG‐based treatment group decreased, this was because GG can capture primitive macrophages by neutralizing scavenger receptors as an anion trap.^[^ ^23^ ^]^ In the BMeGG‐H group, the total number of macrophages (F4/80^+^) was the lowest, reiterating the sustained barrier properties of BMeGG‐H, which ensured intestinal integrity repair, fibrinolysis balance regulation, and caused a positive feedback immune modulation (Figure 9c). The decrease in M1 macrophages (CD 86^+^) in the BMeGG‐H group indicated reduced inflammatory responses (Figure 9a,d), while the decreased M2 macrophages (CD 206^+^) avoided the excessive deposition of fibrous collagen (Figure 9b,e). The downregulation of inflammation and fibrous deposition helped prevent adhesion formation. Despite varying degrees of anti‐inflammatory efficacy in all treatment groups due to the inherent anti‐inflammatory activity of GG,^[^ ^58^ ^]^ the BMeGG‐H group demonstrated the most outstanding immunomodulatory efficacy. This was a comprehensive anti‐adhesion outcome ensured by the synergistic effects of self‐leveling irregular wound adaptability, photo‐controlled instantaneous tissue binding, and asymmetric adhesion barrier to prevent displacement.

The protein and mRNA expression of cytokines was further evaluated to investigate the regulatory effects of BMeGG‐H on the immune response during the adhesion formation phase. The expression levels of TNF‐α and IL‐6, produced by M1 macrophages, were the highest in the model group, followed by the GG, MeGG, Interceed, and BMeGG‐H hydrogel groups, consistent with the trend of adhesion severity (Figure 9f,g). Similarly, TGF‐β1 produced by M2 macrophages showed a consistent trend (Figure 9h). These findings revealed the positive correlation between the downregulation of inflammatory factor expression and the effectiveness of anti‐adhesion treatment with the BMeGG‐H hydrogel.

Furthermore, p65, Smad3, and Smad7 were chosen as representative cytokines involved in inflammation‐induced adhesion pathways to further dissect this aforementioned positive correlation.^[^ ^45^ ^]^ Studies have shown that p65 is a key protein activated by TNF‐α, which can exacerbate inflammation through the NF‐κB pathway, thereby promoting adhesion.^[^ ^59^ ^]^ Meanwhile, Smad3 can be phosphorylated by TGF‐β1 and directly induce the synthesis of extracellular matrix components, thereby promoting fibrous deposition.^[^ ^31a^ ^]^ Furthermore, Smad7 can competitively bind to the TGF‐β type I receptor, thereby preventing the phosphorylation of Smad3 and inhibiting fibrous deposition. The influence of the BMeGG‐H hydrogel on the TNF‐α/P65 and TGF‐β1/Smad signaling pathways was studied through Western blot analysis (Figure 9i). In the model group, p65 and Smad3 protein levels were notably higher than those in the normal group. However, the levels of both proteins were attenuated in all treatment groups, with the extent of attenuation being correlated with therapeutic effectiveness. The expression of Smad7, which is unfavorable for adhesion formation, exhibited the opposite trend in all groups (Figure 9j). These findings further validated that the robust anti‐adhesion efficacy of BMeGG‐H hydrogel could partly be attributed to its role in promoting M2 macrophage polarization, thereby facilitating wound healing and downregulating fibrous deposition through the modulation of the TNF‐α/P65 and TGF‐β1/Smad signaling pathways.

#### 2.7.4 Interaction of the Comprehensive Anti‐Adhesion Routes

**Scheme 3:**
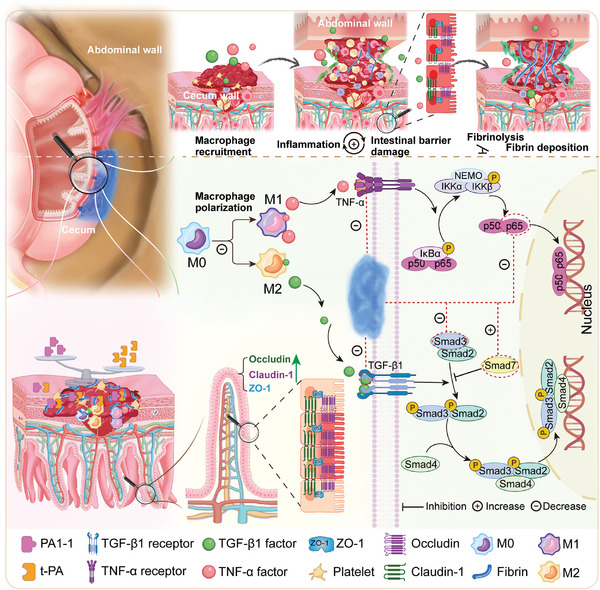
The “three‐in‐one” anti‐adhesion mechanism of BMeGG‐H hydrogel includes maintaining intestinal barrier integrity, regulating fibrosis balance, and reducing the binding of inflammatory factors.

The trinity anti‐adhesion routes of BMeGG‐H discussed above mutually promoted each other, facilitating the healing of postoperative wounds while preventing adhesion occurrence and progression (**Scheme** **3**). Among these pathways, local immune regulation holds a central position. During hemostasis in injured tissue after surgery, the release of thrombin activates t‐PA, triggering the conversion of fibrinogen to fibrin while simultaneously promoting the polarization of resident and exudate macrophages into the pro‐inflammatory M1 phenotype. This eventually results in the inflammatory infiltration of the damaged tissue.^[^ ^60^ ^]^ The cytokine TNF‐α expressed by M1 macrophages promotes inflammation and blood clotting through the NF‐κB signaling pathway.^[^ ^61^ ^]^ Simultaneously, it induces the release of PAI‐1 and inhibits the production of t‐PA activators, hindering fibrinolysis. The cytokine TGF‐β1 expressed by M2 macrophages can modulate the balance of fibrinolysis toward fibrous deposition via the TGF‐β1/Smad3 signaling pathway. The overexpression of IL‐6, TNF‐α, and Smad3, along with the downregulation of TGF‐β1 and Smad7, collectively lead to the development and progression of postoperative adhesions.^[^ ^44^ ^]^ Furthermore, inflammatory infiltration in the intestinal and peritoneal tissue leads to the downregulation of the tight junction proteins Claudin‐1, ZO‐1, and Occludin, exacerbating intestinal wall disruption and promoting inflammation‐induced adhesions.^[^ ^51^ ^]^

Once under the treatment of BMeGG‐H hydrogel, the overexpression of IL‐6 is suppressed, and the abnormal deposition of Col‐1 is eliminated. Additionally, it alleviated local inflammation by suppressing overexpression of the pro‐inflammatory cytokine TNF‐α, reducing t‐PA release, and promoting PAI‐1 production, thereby tilting the fibrinolytic balance toward non‐adhesion. Moreover, BMeGG‐H inhibits the TGF‐β1/Smad3 signaling pathway, activating TGF‐β1/Smad7, effectively suppressing fibrosis. By reducing local inflammatory responses and reinforcing intestinal cell junctions, it broke the vicious cycle of worsening inflammation and intestinal structural damage. In conclusion, BMeGG‐H exhibited outstanding regulation of the fibrinolytic system balance, anti‐inflammatory effects, and improvement of intestinal wall integrity. These three aspects complement each other and collectively achieve effective prevention of postoperative intra‐abdominal adhesions (Scheme 3).

## 3 Conclusion

We developed a customed hydrogel system that overcomes previous challenges of anti‐postoperative adhesion materials including dislocation of anti‐adhesion materials, non‐specific tissue adhesion, and the induction of secondary fibrinolysis disorders. This hydrogel eliminated the limitations of body temperature application of GG‐based hydrogels by introducing methyl acrylate as a “hydrogen bond destroyer” on the molecule under the guidance of theoretical calculations. This hydrogel could self‐level and adapt to irregular wounds ensuring a precise fit at the interface between the hydrogel and injured tissue. Additionally, transient unilateral adhesion could be achieved via light‐operated radical cross‐linking of activated carbon‐carbon double bonds and Thiol‐Ene bonds at the tight‐fitting interfaces. Through comprehensive analysis at the genetic, molecular, and tissue levels, our hydrogel system has been shown to regulate fibrinolysis balance, suppress self‐inflammatory responses, restore intestinal wall integrity, and adjust the t‐PA/PAI‐1 balance, ultimately leading to a remarkable anti‐adhesion effectiveness of 100% over a 14‐day period. This efficacy significantly surpasses the 85% effectiveness observed with current clinical materials. These findings underscore the potential of our hydrogel system as a promising solution for preventing postoperative adhesion, offering new avenues for enhancing patient outcomes and improving surgical interventions.

## 4 Experimental Section

### Animal Ethics Statement

Male SD rats (220 ± 20 g) were obtained from the Animal Center of the Air Force Medical University. Animals were housed in IVC‐grade animal facilities. All animal experiments were approved by the Animal Care and Use Committee of Northwest University (License Number: NWU‐AWC‐20220904 M).

### Statistical Analysis

Each sample was tested at least three times, and the results were expressed as mean ± standard deviation. Material simulations were performed using Materials Studio 2020 software. Statistical analysis was performed using software including GraphPad Prism, Origin, SPSS, and Image J. Adhesion scores did not always follow a normal distribution, so a non‐parametric Mann‐Whitney U test was used for statistical analysis. Body weight data were normally distributed and were analyzed using a one‐way analysis of variance followed by Tukey's multiple comparison test. A 95% confidence level was used for all analyses, and two‐tailed tests were employed. Statistical significance was defined as ^*^ *p* < 0.05, ^**^ *p* < 0.01, and ^***^ *p* < 0.001 was considered significant.

## Conflict of Interest

The authors declare no conflict of interest.

## Supporting information

Supporting Information

Supplemental Movie 1

Supplemental Movie 2

## Data Availability

The data that support the findings of this study are available from the corresponding author upon reasonable request.
