# Supplementary material for: Light‐Operated Transient Unilateral Adhesive Hydrogel for Comprehensive Prevention of Postoperative Adhesions
Source: Adv Sci (Weinh). 2024 Jun 25;11(32):2403626. doi: 10.1002/advs.202403626 (PMC11348232; doi:10.1002/advs.202403626)
Supplement: Supplementary file 1 — Supporting Information [file ADVS-11-2403626-s002.docx]

**Supporting** **Information**

**Light-Operated Transient Unilateral Adhesive Hydrogel for Comprehensive Prevention of Postoperative Adhesions**

*Furong Cui^1,2,3^, Shihong Shen^1,2,3*^, Xiaoxuan Ma^1,2,3^, Daidi Fan^1,2,3*^*

F. R. Cui, Prof. S. H. Shen, Prof. X. X. Ma, Prof. D. D. Fan,

1. Engineering Research Center of Western Resource Innovation Medicine Green Manufacturing, Ministry of Education, School of Chemical Engineering, Northwest University, Xi'an, 710069, China

2. Shaanxi Key Laboratory of Degradable Biomedical Materials and Shaanxi R&D Center of Biomaterials and Fermentation Engineering, School of Chemical Engineering, Northwest University, Xi'an, 710069, China

3. Biotech. & Biomed. Research Institute, Northwest University, Xi'an, 710069, China

E-mail: shihongshen@nwu.edu.cn

E-mail: fandaidi@nwu.edu.cn

Tel: 086-029-88305118; Fax: 086-029-88322585

ORCID ID

Shihong Shen ID (0000-0003-1448-9823)

Daidi Fan ID (0000-0001-9798-1674)

# Keywords: unilateral tissue adhesion, light-operated, hydrogen bond-modulated hydrogel, anti-postoperative adhesion

# 1 Experimental Section

## 1.1 Materials

Gellan Gum (GG, Mw = 10^6^), methacrylic anhydride (MA, ≥94%), and boric acid (≥99.5%) were purchased from Sigma-Aldrich (Shanghai, China). Reagents used in the cell culture experiments were purchased from Solarbio (Beijing, China). The Col-1 protein analysis kit was sourced from Boster Biological Technology Co., Ltd. (Wuhan, China). Fibrinogen was purchased from Sigma-Aldrich (Shanghai, China). All primary antibodies used in the experiments were purchased from Proteintech (Wuhan, China), whereas all secondary antibodies were purchased from Abbkine (Wuhan, China). The TaqMan™ reverse transcription kit and Fast SYBR™ Green Master Mix were supplied by Thermo Fisher Scientific (Xi'an, China). ELISA kits were obtained from Shanghai Enzyme-linked Biotechnology Co., Ltd. (Shanghai, China).

## 1.2 Cells

The mouse embryonic fibroblast cell line (L929) was purchased from Wuhan Pusen Life Science and Technology Co., Ltd. The blood cells were obtained from normal Sprague-Dawley (SD) rats.

## 1.3 Animals

Male SD rats (220 ± 20 g) were obtained from the Animal Center of the Air Force Medical University. Animals were housed in IVC-grade animal facilities (License Number: Shaanxi 2015-002). This study involving animal work was approved by the Animal Care and Use Committee of Northwest University (License Number: NWU NWU-AWC-20220904M).

## 1.4 Synthesis of BMeGG

MeGG was obtained according to the previous report.^[1]^ GG (1g) was dissolved in 100mL of deionized water. After stirring at 90 °C for one hour, the solution was cooled to 50 °C. Varying amounts of methacrylic anhydride (MA) were added to the GG solution, and 0.1M NaOH was used to adjust the pH of the system to maintain a pH of 8.0 during the reaction. After 12 hours of reaction, the product was collected for dialysis, and MeGG foam was obtained after freeze-drying. BMeGG was formed by complexing MeGG with borax. MeGG with different degrees of MA grafting was dissolved in 0.1M borate buffer solution, and then freeze-dried to obtain the borate-glycol complex of MeGG, which is BMeGG. Purified BMeGG was obtained by lyophilization and stored in a dry place, avoiding light.

## 1.5 Preparation of BMeGG-H

First, a 2 wt% BMeGG precursor solution was prepared, and varying amounts of I2959 (photo-initiator) were added to the solution. Under 365 nm ultraviolet irradiation, BMeGG-H was formed. The gelation time of the hydrogel was tested using a rotational rheometer (MCR302, Anton-Paar) at 25 °C and 37 °C.

## 1.6 Characterization

The chemical structure of BMeGG-H was analyzed using proton nuclear magnetic resonance (1H NMR) spectroscopy with a Varian Inova 500 NMR spectrometer equipped with a variable temperature system. Lyophilized materials were dissolved in D_2_O at a concentration of 10 mg/mL and at a temperature of 50 °C. Fourier transform infrared spectra (FT-IR) (IRAffinity-1S) were acquired in the range of 500-4000 cm^−1^ to analyze the chemical bonds present in BMeGG-H. The grafting degree was calculated based on previous research.^[2]^ The resolution was set at 4 cm^−1^. X-ray diffraction analyses were conducted on GG, MeGG with 10% MA degree grafting GD), and MeGG with 10% MA GD hydrogels using an X-ray diffractometer (Mini Flex600) with a scanning speed of 0.04 degrees per second and a diffraction angle (2θ) range of 5 to 40 degrees. Following freeze-drying, the hydrogel samples underwent sputter-coating with a thin layer of gold within a vacuum chamber. Subsequently, the cross-sectional and surface morphologies of the samples were examined using a field emission scanning electron microscope (SEM, N7000, Zeiss).

## 1.7 Rheology

For rheological testing, solutions consisting of 2 wt% GG with 0.1 wt% I2959, as well as 2 wt% MeGG and BMeGG, were dispensed into cylindrical molds (Height × Diameter: 1 mm × 20 mm). Each test was conducted with three samples. Experiments were performed utilizing a rheometer (MCR302, Anton-Paar) equipped with a 20 mm diameter parallel-plate geometry. Initially, a frequency sweep ranging from 0.1 to 10 Hz was performed at a strain of 1% to assess the storage modulus (G') and loss modulus (G'') of the hydrogels. Subsequent oscillatory strain sweep tests were conducted over a range of shear strains from 1 to 100% at a frequency of 1 Hz. Furthermore, the steady shear viscosity of the BMeGG-H precursor was determined by varying the shear rate from 0.1 to 10 s^−1^. Dynamic rheological analyses were conducted using a MCR302, Anton-Paar rheometer equipped with a 20 mm diameter parallel-plate geometry. Time-sweep oscillatory tests of the BMeGG-H hydrogel were performed with a 0.5 mm gap, while the strain and frequency were set at 1% and 1 Hz, respectively. Temperature sweep tests were conducted over a temperature range of 20 to 55 °C to assess thermosensitive behavior, with a heating rate of 1 °C/min. Each experiment was performed in triplicate.

## 1.8 Injectable and self-leveling

To test the injectability and self-leveling properties of BMeGG, a 2% BMeGG (GD = 10%) solution was loaded into a syringe and extruded through a 7-gauge needle to form the letters "NWU". Similarly, BMeGG was injected into a star-shaped mold and, after 10 minutes, adapted to form a star-shaped gel. The gel injected onto glass beads demonstrated self-leveling properties, forming a complete covering layer over the glass beads.

## 1.9 Molecular dynamics simulations with hydrogen bond modulation

Based on previous study.^[3]^ Initially, the DMOL3 module in Materials Studio 2020, utilizing density functional theory, was employed for the structural optimization and atomic charge calculations (ESP method) of both monomer and grafted monomer molecules. A chain model with a polymerization degree of 5 was constructed, and grafting densities of 0, 2 (head-tail), 5, and 10 chains were situated in orthogonal boxes. Amorphous unit cell models with grafting densities of 0, 2, and 5 were constructed for molecular dynamics simulations. The total simulation time amounts to 2 ns, comprising 1 ns of NPT (isothermal-isobaric) and 1 ns of NVT (isothermal-isovolumetric) simulations. According to previous reports, Radial Distribution Function (RDF) was set up.^[4]^ The final 100 structures are utilized to calculate the RDF, depicting the probability density of the occurrence of another atom around a central atom based on the interaction distance between two atoms. A higher RDF implies a stronger interaction between the two atoms.

## 1.10 Photo-controlled unilateral adhesion of proteins, cells, and tissues

*In vitro* adhesion experiments referenced previous report.^[5]^ Initially, sterilized BMeGG was used to coat each well of a 24-well plate, followed by crosslinking using a 365 nm UV light. Subsequently, BMeGG-H hydrogel samples were immersed in 2 mL of DMEM culture medium containing 10% FBS under standard culture conditions at 37 °C and 5% CO_2_ for 2 hours. After incubation, the medium was aspirated, and 2 mL of fresh medium containing fibroblasts (L929 cells) at a concentration of 3×10^4^ cells per plate, or rhodamine-labeled fibrinogen, or blood cells was dropwise added onto the surface of the BMeGG-H hydrogel or the 24-well cell culture plates (tissue culture polystyrene, TCP). Cells cultured on TCP served as the control group. The cells were then cultured for 48 hours, after which the medium was aspirated, and the cells were washed with PBS. Following washing, L929 cells were observed under a microscope, and fibrinogen was observed using fluorescence microscopy (Olympus), while blood cell adhesion was observed using electron microscopy.

## 1.11 Anti-adhesion efficacy in vitro

The hydrogels from each group were co-cultured with LPS-induced macrophages. Every two days, the remaining hydrogels were transferred to new cell culture dishes with the same cell number and density. The concentrations of TNF-α, IL-6, and TGF-β1 in the culture medium were measured to evaluate the impact of hydrogel degradation and dissolution products on macrophages.

## 1.12 In vivo degradation and host-guest interaction

The establishment of subcutaneous degradation models was based on previous report.^[6]^ BMeGG-H hydrogels were fabricated through photo-crosslinking and subsequently subcutaneously implanted into 6-week-old male C57BL/6 mice which were randomly divided into groups.. At predetermined intervals (2 weeks post-implantation), major organs including the heart, liver, spleen, lung, and kidney were harvested. Pathological changes were observed using hematoxylin and eosin (H&E) staining. The hemolytic potential of BMeGG-H hydrogels was evaluated by incubating them with mouse red blood cells.^[7]^ Initially, pericardial blood from Sprague Dawley (SD) rats was collected in EDTA-containing blood collection tubes. The blood was gently mixed and centrifuged at 3000 rpm for 5 min at 4 °C. After removing the serum, an equal volume of freshly prepared 0.9% saline solution was added. This washing process was repeated five times to ensure complete removal of residual serum. Subsequently, red blood cells (RBCs) were diluted with 0.1 M PBS to obtain a 1×10^8^ mL^−1^ RBC suspension. The BMeGG-H hydrogel samples were then incubated with the RBC suspension. Negative and positive controls were prepared using saline solution and DD water, respectively. After 120 minutes of incubation at 37 °C, the suspension was centrifuged for 5 min at 2000 rpm. The supernatant was transferred to a 96-well plate, and the absorbance was measured at 541 nm using a microplate reader (BioTek,800 TS). The hemolysis rate was calculated using the formula: $\begin{aligned} \text{Hemolysis rate\%=}\frac{\text{As-AC}}{\text{At}}\text{×100\%}\text{ }\left( \text{1} \right) \end{aligned}$

Where As, Ac, and At represent the absorbance at 541 nm for the sample group, saline solution group, and DD water group, respectively.

## 1.13 In vivo anti-adhesion efficacy

Animal experiments were conducted following the guidelines of the experimental animal administration committee of Northwest University. The establishment of the animal model was based on previous report.^[8]^ Adult male SD rats (n=30, 200-250 g) were randomly allocated into five groups: Normal (no surgery), Model, GG, and BMeGG-H hydrogel groups. Prior to surgery, animals were anesthetized by intraperitoneal injection of 1% sodium pentobarbital (40 mg/kg). Following anesthesia, the abdominal wall was shaved and disinfected. A 4 cm midline incision was then made to expose the abdomen and cecum. Subsequently, the cecum was abraded using a surgical brush to create a petechial hemorrhage area (1.5 cm × 1.5 cm) on its surface. Following this, a peritoneal defect measuring 1.5 cm × 1.5 cm corresponding to the petechial hemorrhage on the cecum was created using a scalpel. Following this, the two injured surfaces were apposed using 3-0 silk sutures to ensure firm contact. The Model group received a 1 mL injection of physiological saline into the abdominal cavity. The GG group was treated with 1 mL of Medical Gellan Gum Gel. Rats that received no treatment were assigned to the Normal group. Finally, the abdomen and skin were closed sequentially with 4-0 surgical silk sutures. All procedures were conducted meticulously by a skilled surgeon and adhered strictly to aseptic principles. Postoperatively, rats were administered penicillin at a dose of 1×10^5^ U per rat for 3 days to prevent infection and fasted for 2 days. Animal weights were monitored every three days to assess gastrointestinal motility recovery.

Adhesion tissues on postoperative Day 7 were observed using a double-blind method, and scoring criteria were based on previous report.^[9]^

## 1.14 Evaluation of Fibrinolysis Regulation by BMeGG-H

After 7 days post-surgery, we observed the expression levels of t-PA, PAI-1, and Col-1 in the model tissues through immunofluorescence staining. The method followed previous study.^[10]^ Subsequently, we conducted semi-quantitative analysis of relative fluorescence intensity using Image J software. Additionally, at the gene level, quantitative analysis of mRNA transcription levels of t-PA, PAI-1, and Col-1 in the aforementioned tissues was performed using reverse transcription-polymerase chain reaction (RT-PCR).

## 1.15 Observation and Analysis of Intestinal Wall Integrity

We observed the microstructural changes in the modeling sites of each group through staining with HE, Masson, PAS, and Alcian blue. Additionally, we quantitatively analyzed the expression levels and transcriptional quantities of tight junction proteins ZO-1, Claudin-1, and occludin at both the protein (using Western blotting, WB) and gene (using reverse transcription-polymerase chain reaction, RT-PCR) levels. All the RT-qPCR primers sequences were listed in **Table S7**.

## 1.16 The Immunomodulatory Capability Assessment of BMeGG-H

Perform immunofluorescence staining for analyzing the M1-to-M2 phenotype transition of macrophages. Use ELISA kits following the manufacturer's instructions to measure the expression levels of interleukin-6 (IL-6), tumor necrosis factor alpha (TNF-α), and transforming growth factor beta 1 (TGF-β1) in rat serum. Quantify the expression levels of Smad3, Smad7, and p65 in the modeling site tissues through Western blot (WB) analysis.

## 1.17 Statistical analysis

Each sample was tested at least three times, and the results were expressed as mean ± standard deviation. Material simulations were performed using Materials Studio 2020 software. Statistical analysis was performed using software including GraphPad Prism, Origin, SPSS and Image J. Adhesion scores did not always follow a normal distribution, so non-parametric Mann-Whitney U test was used for statistical analysis. Body weight data were normally distributed and were analyzed using one-way analysis of variance followed by Tukey's multiple comparison test. A 95% confidence level was used for all analyses, and two-tailed tests were employed. Statistical significance was defined as **p* < 0.05, ***p* < 0.01, and ****p* < 0.001 was considered significant.

# 2 Supplementary Experimental Results

**Table S1.** Preparation of GG with different degrees of grafting by reacting with varying amounts of methacrylic anhydride.

| GG Methacrylate | GG(g) | Methacrylic anhydride(mL) | Degree of substitution (%) | |
| --- | --- | --- | --- | --- |
| Low GD | 1 | 4 | | 7.39±0.76 |
| Medium GD | 1 | 8 | | 19.31±1.02 |
| High GD | 1 | 12 | | 25.33±1.47 |

**Table S2ffect of different borax contents on 4 cm2 self-leveling area.**

| GG Methacrylate | Borax (wt%) | Injection volume(mL) | Self-leveling time (s) | |
| --- | --- | --- | --- | --- |
| Low GD | 0.75% | 0.3 | | 60 ± 5 |
| Low GD | 1.00% | 0.3 | | 77 ± 4 |
| Low GD | 1.50% | 0.3 | | 130 ± 17 |
| Low GD | 1.75% | 0.3 | | 190 ± 15 |
| Medium GD | 0.75% | 0.3 | | 24 ± 2 |
| Medium GD | 1.00% | 0.3 | | 31 ± 4 |
| Medium GD | 1.50% | 0.3 | | 35 ± 3 |
| Medium GD | 1.75% | 0.3 | | 49 ± 6 |
| High GD | 0.75% | 0.3 | | 11 ± 1 |
| High GD | 1.00% | 0.3 | | 15 ± 3 |
| High GD | 1.50% | 0.3 | | 21 ± 5 |
| High GD | 1.75% | 0.3 | | 32 ± 6 |


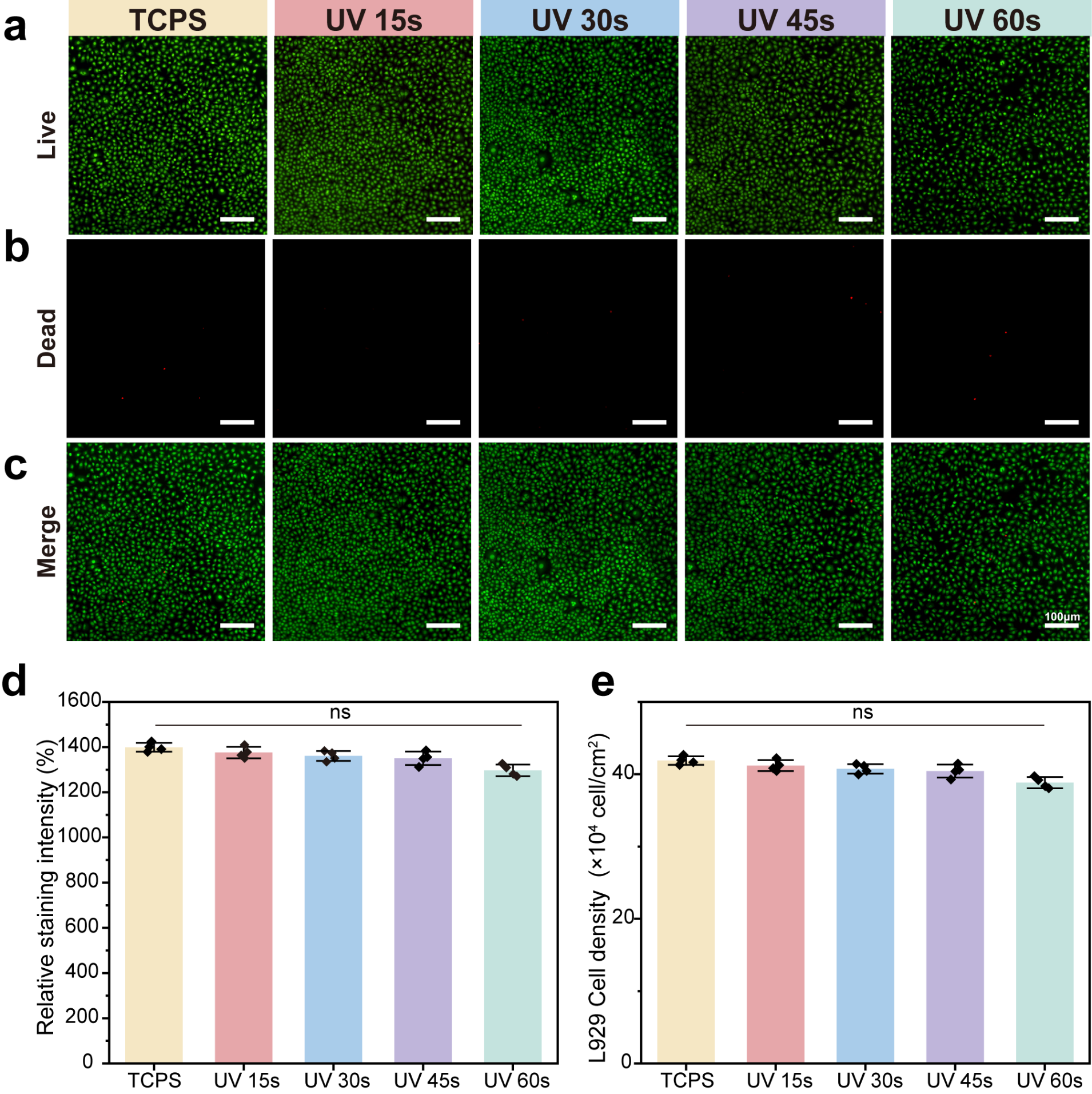


**Figure S1.** a-c) Cytocompatibility in Live/Dead assay. After UV irradiation for different times at a light irradiance density of 7.14 mW/cm², mouse fibroblasts (L929) were cultured for 24 hours. Scale = 100 µm. d) Fluorescence intensity of living L929 cells in each group. e) Number of viable L929 cells in each group.


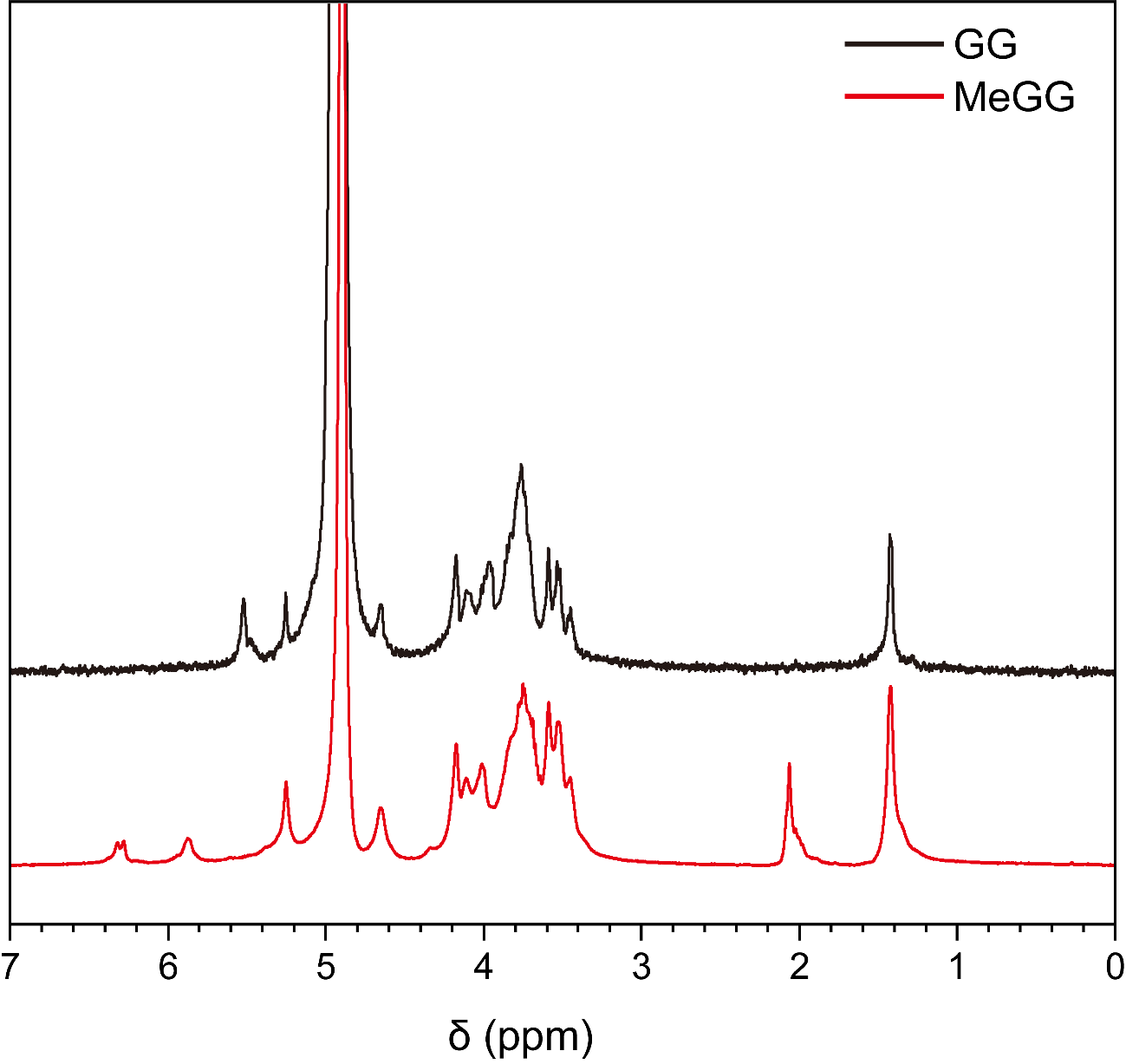


**Figure S2.** ^1^H NMR spectra of (B) GG and MeGG recorded in D_2_O (T = 50 °C). The methyl group of MA is located at δ 2.09 ppm, and the vinyl groups of MA are identified around δ 5.5-7 ppm.

$$\begin{aligned} \text{G}\text{D=}\frac{{\frac{\text{I}_{\text{DB}}}{\text{n}_{\text{H}_{\text{DB}}}}}/{\frac{\text{I}_{\text{C}\text{H}_{\text{3rham}}}}{\text{n}_{\text{H}_{\text{C}\text{H}_{\text{3}}\text{rham}}}}}}{\text{n}_{\text{OH}_{\text{m}\text{onomer}}}}\#\left( \text{2} \right) \end{aligned}$$

**Equation S1.** The grafting degree (GD) of MA on GG (fraction of modified hydroxyl groups per repeating unit) was determined by the relative integration of the double bond proton peak (*I*_DB_) of the methacrylate groups to the methyl protons of the internal standard *I*_CH3rham_. Where nH_DB_ and nH_CH3rham_ correspond to the number of protons in the double bond and the methyl group of rhamnose, respectively. nOH_monomer_ corresponds to the number of reactive hydroxyl group in the GG structure.

**Table S3.** Intramolecular hydrogen bond lengths and average hydrogen bond length of GG and MeGG.

| Model | H-bonds | Bonding Length (Å) | Model | H-bonds | Bonding Length (Å) |
| --- | --- | --- | --- | --- | --- |
| GG | H-bond 0 | 2.270317 | MeGG | H-bond 0 | 2.335003 |
|  | H-bond 1 | 2.246574 |  | H-bond 1 | 1.798735 |
|  | H-bond 2 | 1.703601 |  | H-bond 2 | 2.495971 |
|  | H-bond 3 | 1.922012 |  | H-bond 3 | 2.378975 |
|  | H-bond 4 | 1.808929 |  | H-bond 4 | 2.221167 |
|  | H-bond 5 | 1.740036 |  | H-bond 5 | 2.330448 |
|  | H-bond 6 | 2.479795 |  | H-bond 6 | 2.431427 |
|  | H-bond 7 | 2.326078 |  | H-bond 7 | 2.355443 |
|  | H-bond 8 | 2.468229 |  | H-bond 8 | 2.412759 |
|  | H-bond 9 | 2.429243 |  | H-bond 9 | 2.161699 |
|  | H-bond 10 | 1.672435 |  | H-bond 10 | 2.458143 |
|  | H-bond 11 | 1.919284 |  | H-bond 11 | 2.301107 |
|  | H-bond 12 | 2.35672 |  | H-bond 12 | 2.39201 |
|  | H-bond 13 | 2.246928 |  | H-bond 13 | 1.934616 |
|  | H-bond 14 | 2.375002 |  | H-bond 14 | 2.131366 |
|  | H-bond 15 | 2.443148 |  | H-bond 15 | 2.368282 |
|  | H-bond 16 | 1.63412 |  | H-bond 16 | 2.015321 |
|  | H-bond 17 | 1.995278 |  | H-bond 17 | 2.349325 |
|  | H-bond 18 | 1.945966 |  | H-bond 18 | 2.084274 |
|  | H-bond 19 | 1.60782 |  | H-bond 19 | 2.3547 |
|  | H-bond 20 | 2.297013 |  | H-bond 20 | 2.289518 |
|  | H-bond 21 | 2.392815 |  | H-bond 21 | 2.233859 |
|  | H-bond 22 | 2.315634 |  | H-bond 22 | 1.809829 |
|  | H-bond 23 | 2.372197 |  | H-bond 23 | 1.739751 |
|  | H-bond 24 | 2.490876 |  | H-bond 24 | 2.377543 |
|  | H-bond 25 | 2.417677 |  | H-bond 25 | 1.84783 |
|  | H-bond 26 | 2.343222 |  | H-bond 26 | 2.40364 |
|  | H-bond 27 | 2.275314 |  | H-bond 27 | 2.429789 |
|  | H-bond 28 | 2.326881 |  | Average | 2.23009 |
|  | H-bond 29 | 2.303744 |  |  |  |
|  | H-bond 30 | 2.414246 |  |  |  |
|  | H-bond 31 | 2.336533 |  |  |  |
|  | H-bond 32 | 2.35857 |  |  |  |
|  | H-bond 33 | 2.451583 |  |  |  |
|  | H-bond 34 | 2.448338 |  |  |  |
|  | H-bond 35 | 2.224024 |  |  |  |
|  | H-bond 36 | 2.285959 |  |  |  |
|  | H-bond 37 | 2.271302 |  |  |  |
|  | H-bond 38 | 2.386143 |  |  |  |
|  | Average | 2.212912 |  |  |  |

**
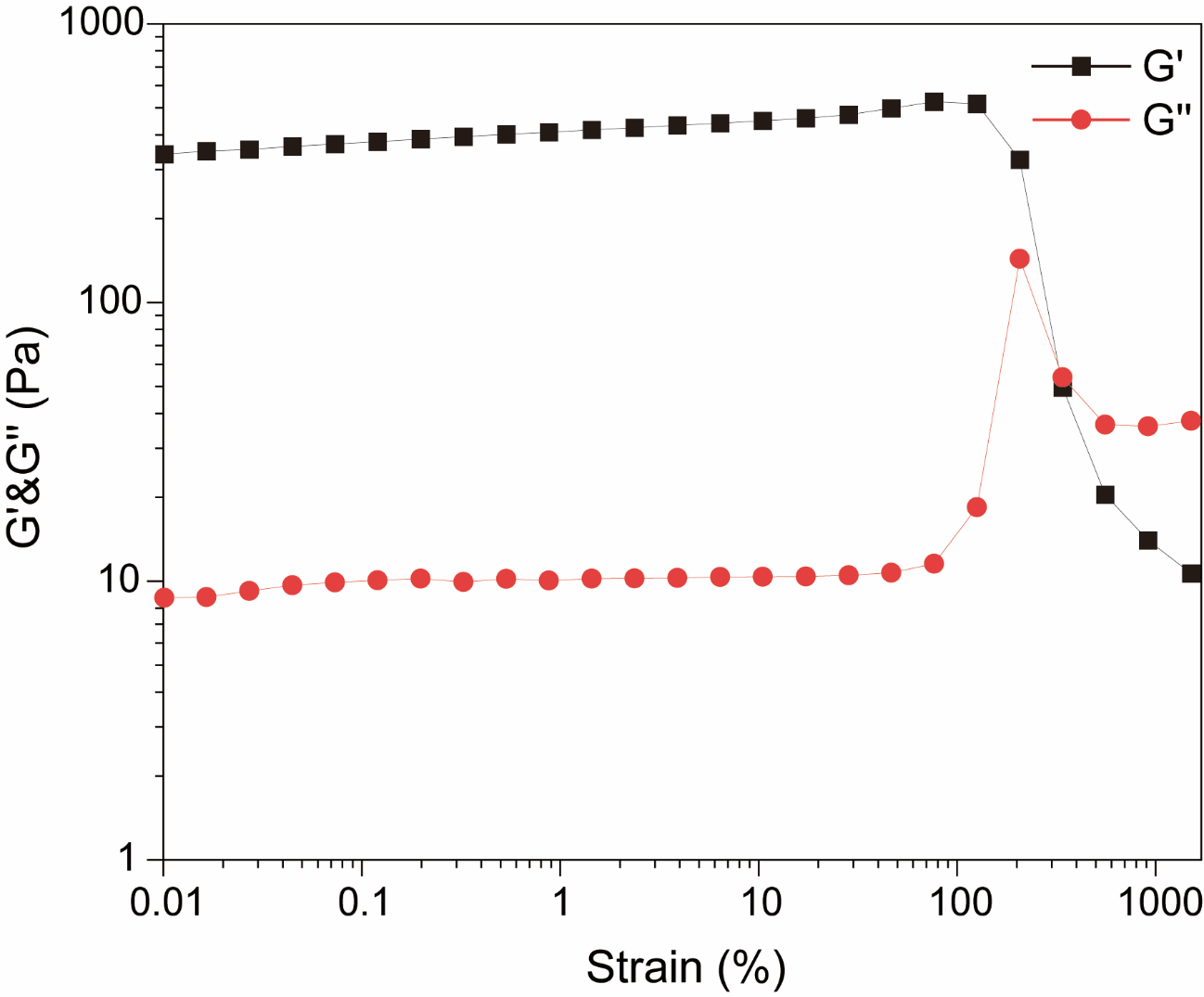
**

### **Figure S3.** G' and G'' of BMeGG with 25% MA grafting under strain amplitude (1 Hz, 37 °C) scanning.


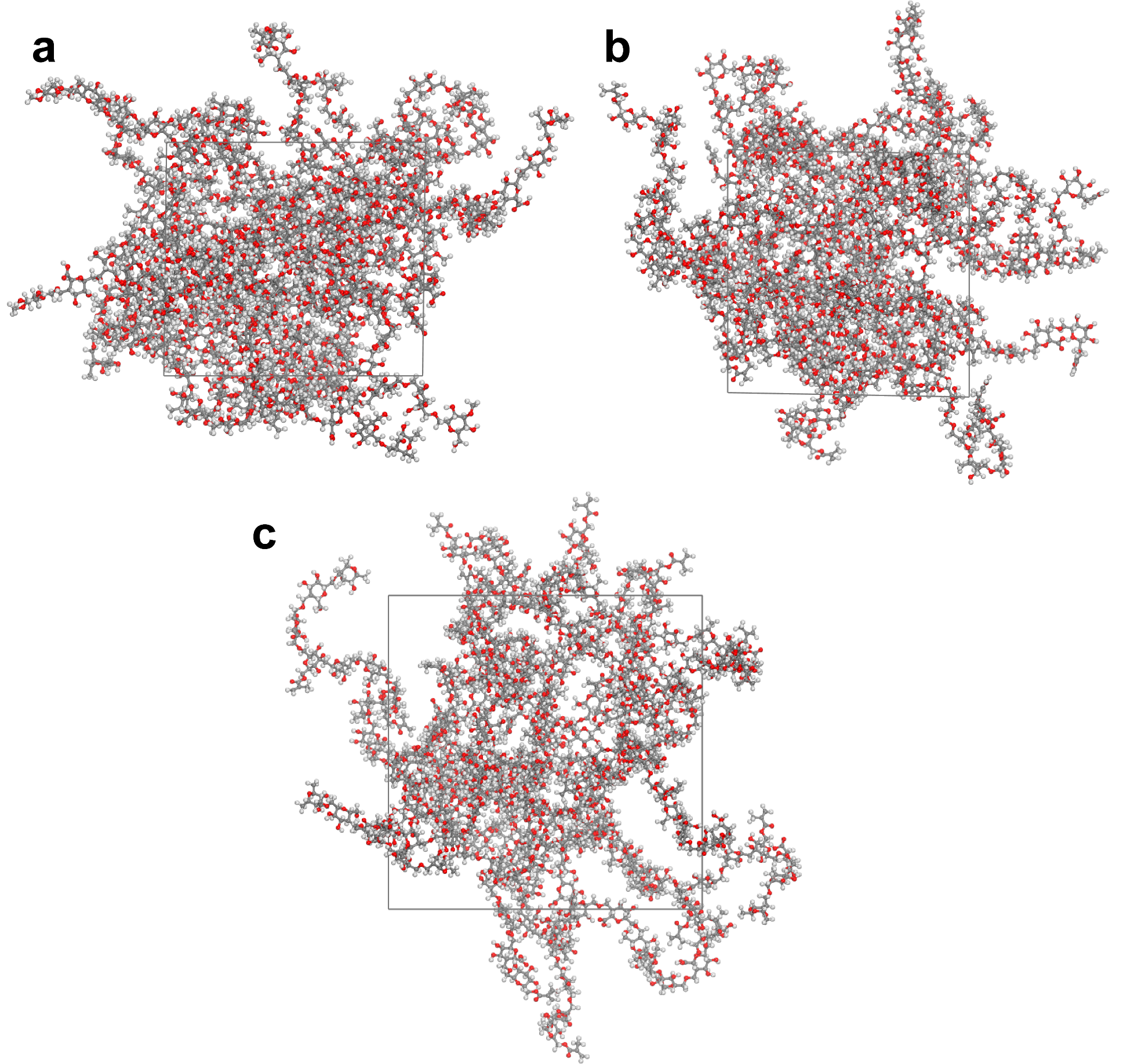


**Figure S4.** a), b), and c) box models represent polymer models with degrees of polymerization (DP) of 5 and a total chain number of 10. These models correspond to without grafted (0% degree grafting of MeGG), grafted at both ends (10% grafting degree of MeGG), and grafted with 5 branches (25% grafting degree of MeGG), respectively.


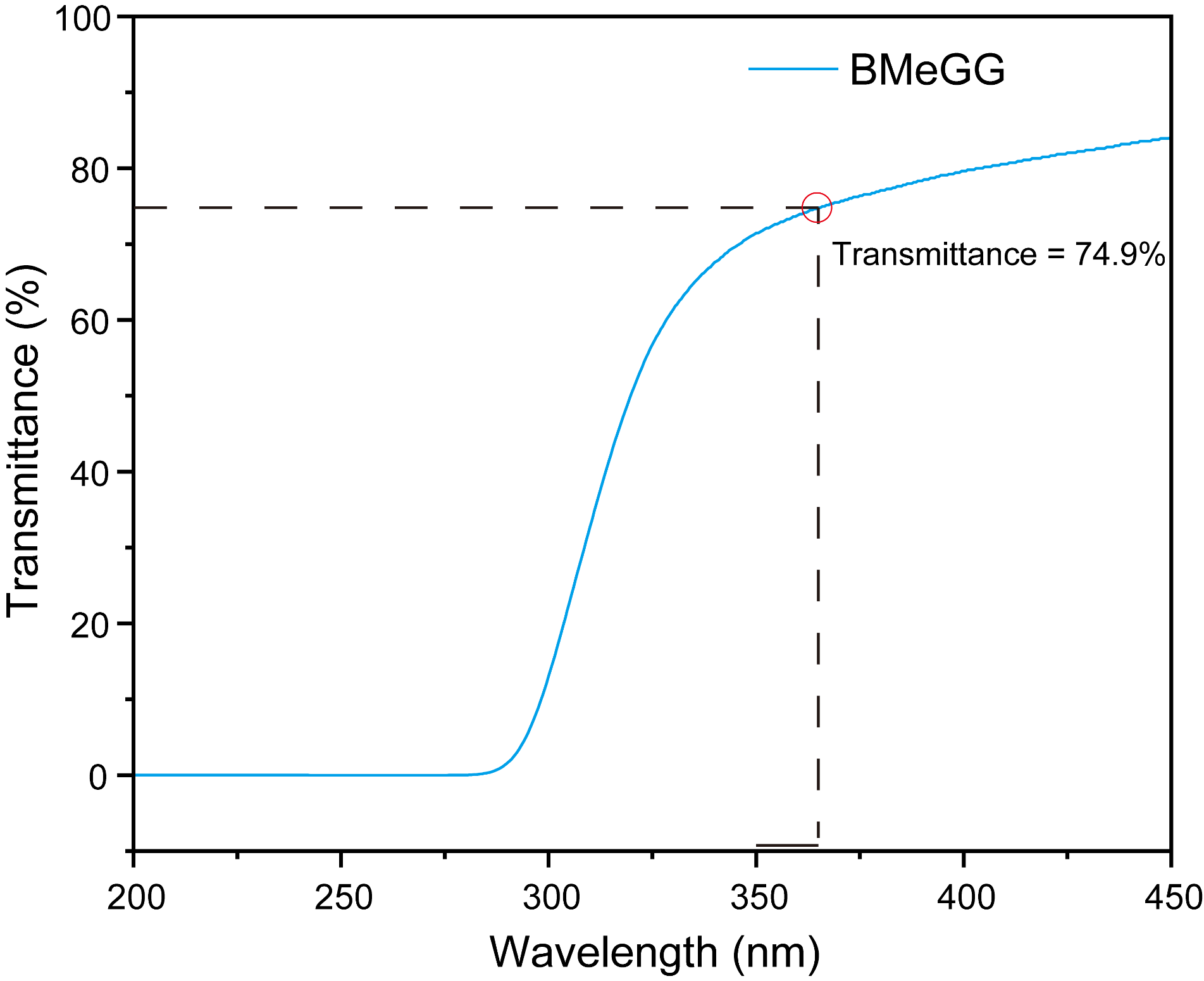


**Figure S5.** The transmittance of 1-2 mm thick BMeGG in the 200-450 nm range..


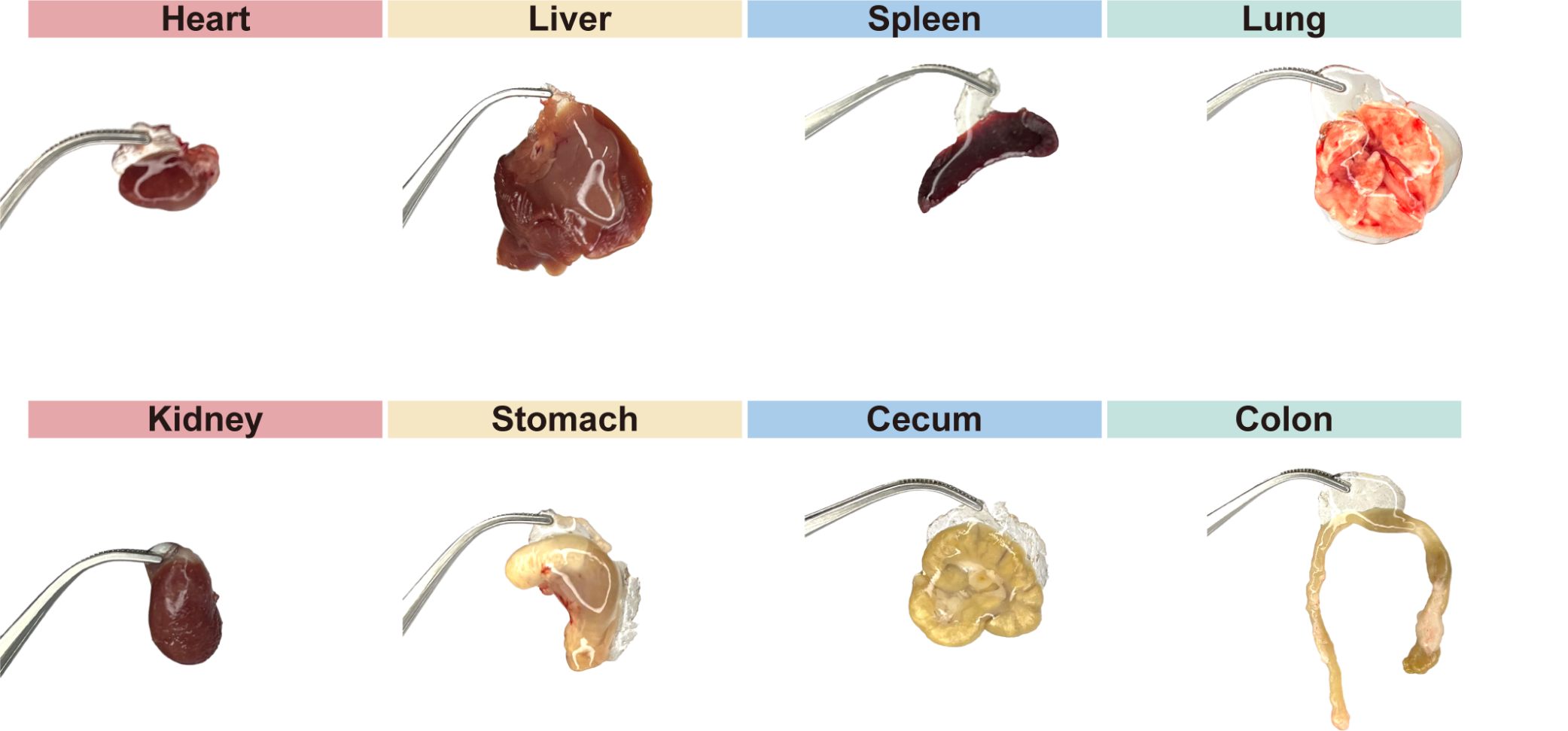


**Figure S6.** Images of photo-controlled instantaneous unilateral adhesion of BMeGG-H on the surfaces of different organs (heart, liver, spleen, lung, kidney, stomach, cecum, colon).


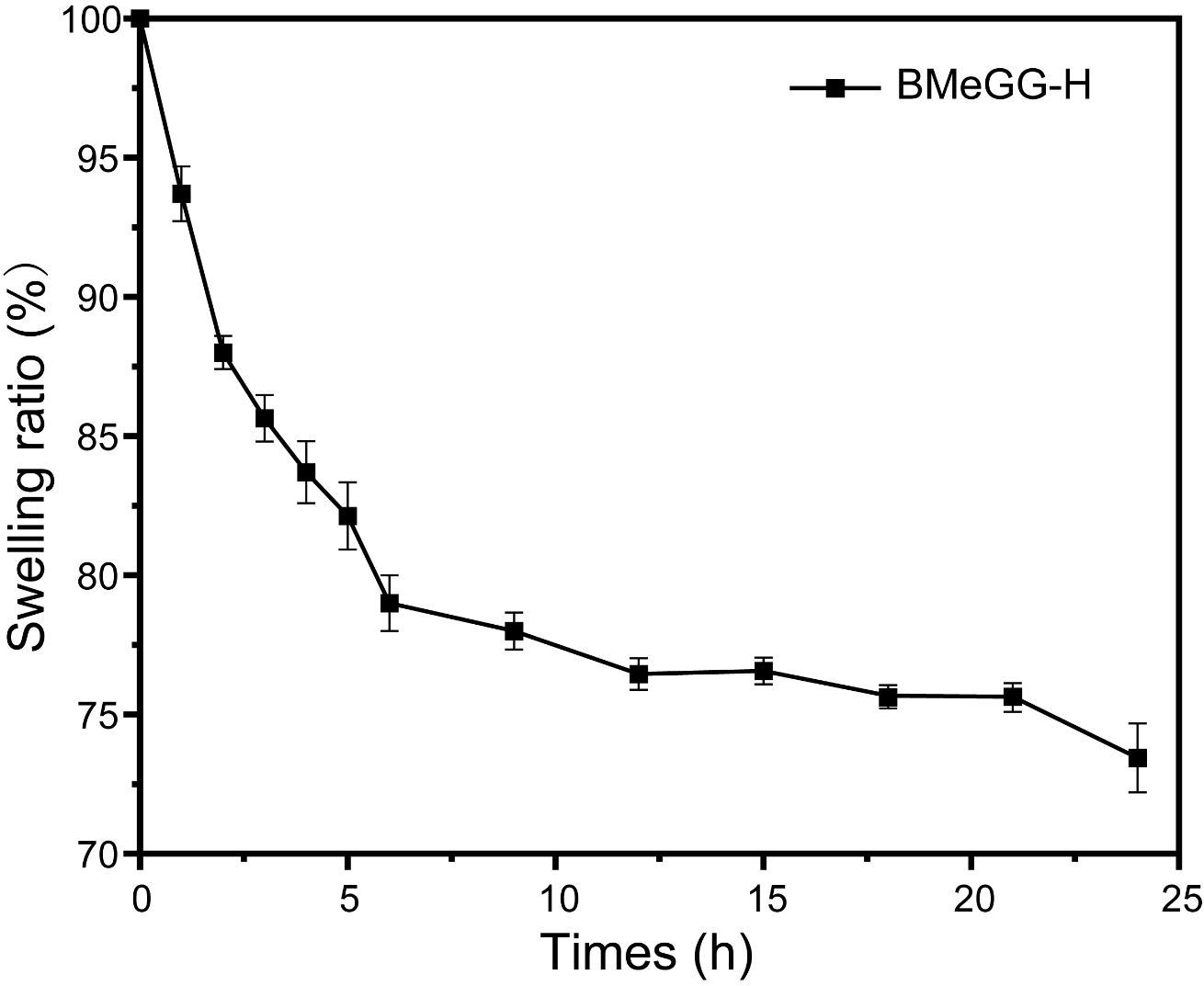


**Figure S7.** Swelling ratio of BMeGG-H in PBS at 37 degrees Celsius, with a testing duration of 24 hours.


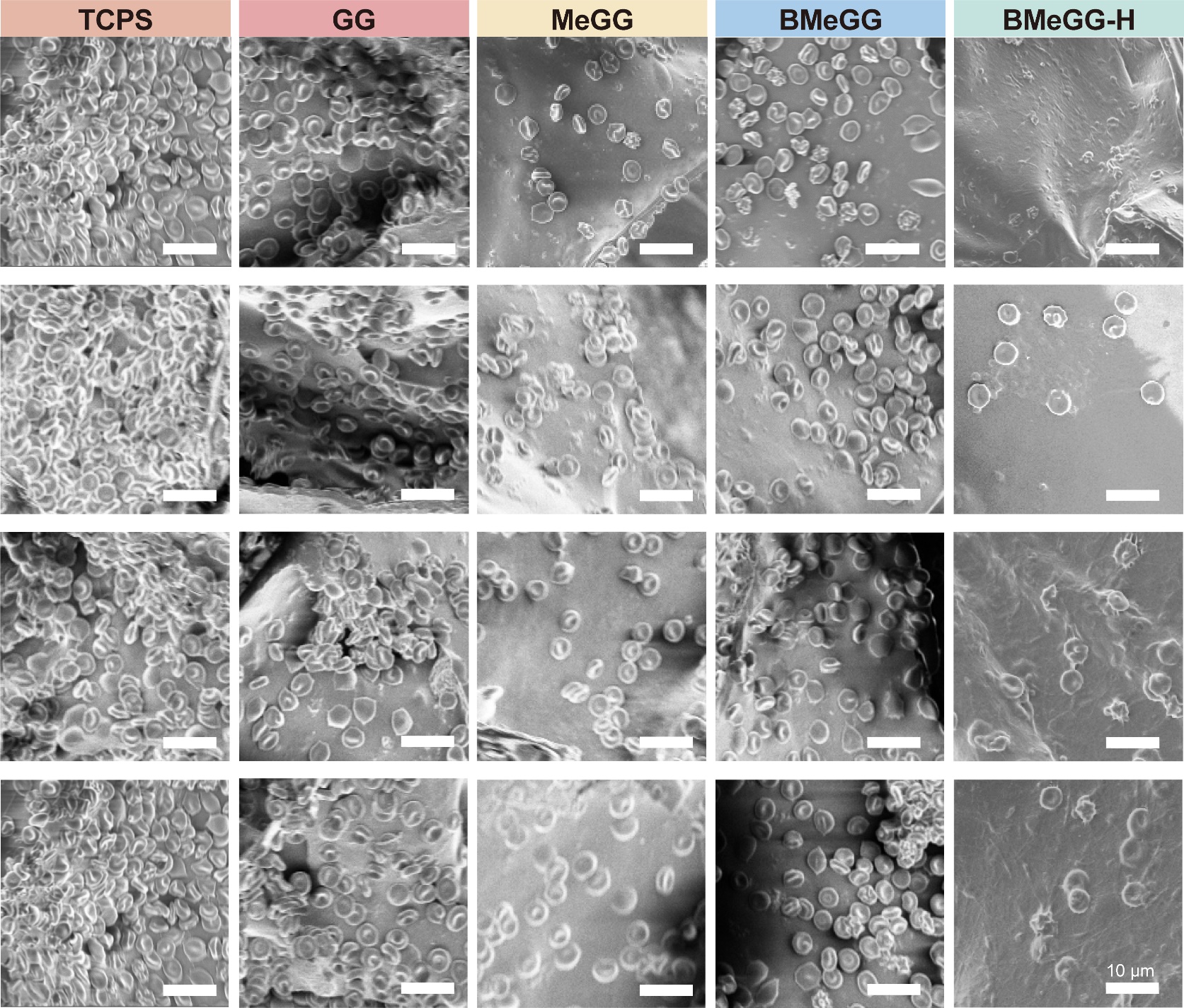


**Figure S8.** All the images used for the statistical analysis of blood cell adhesion in each group.


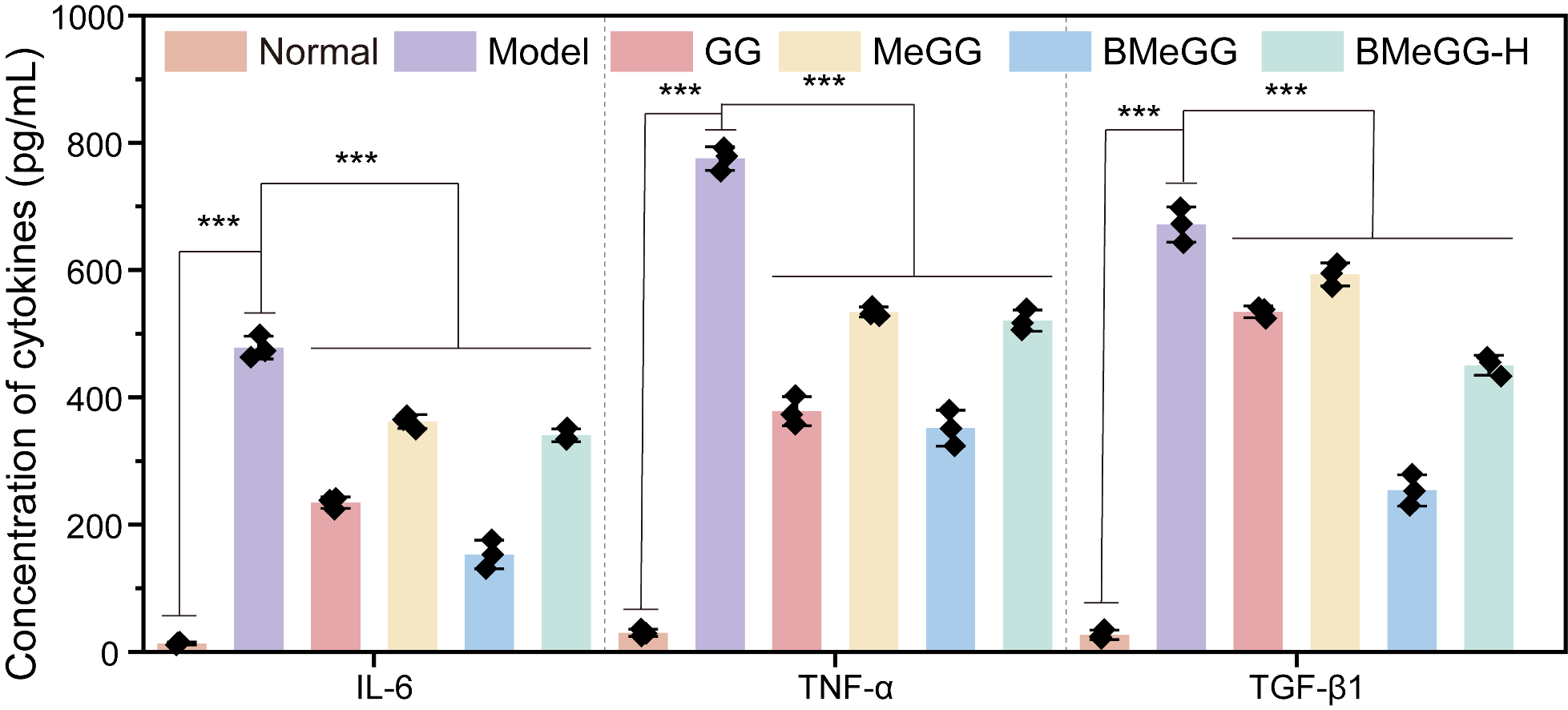


**Figure S9.** The expression levels of TNF-α, TGF-β1, and IL-6 in RAW264.7 macrophages were measured after co-culturing with four types of GG-based hydrogels for one day.


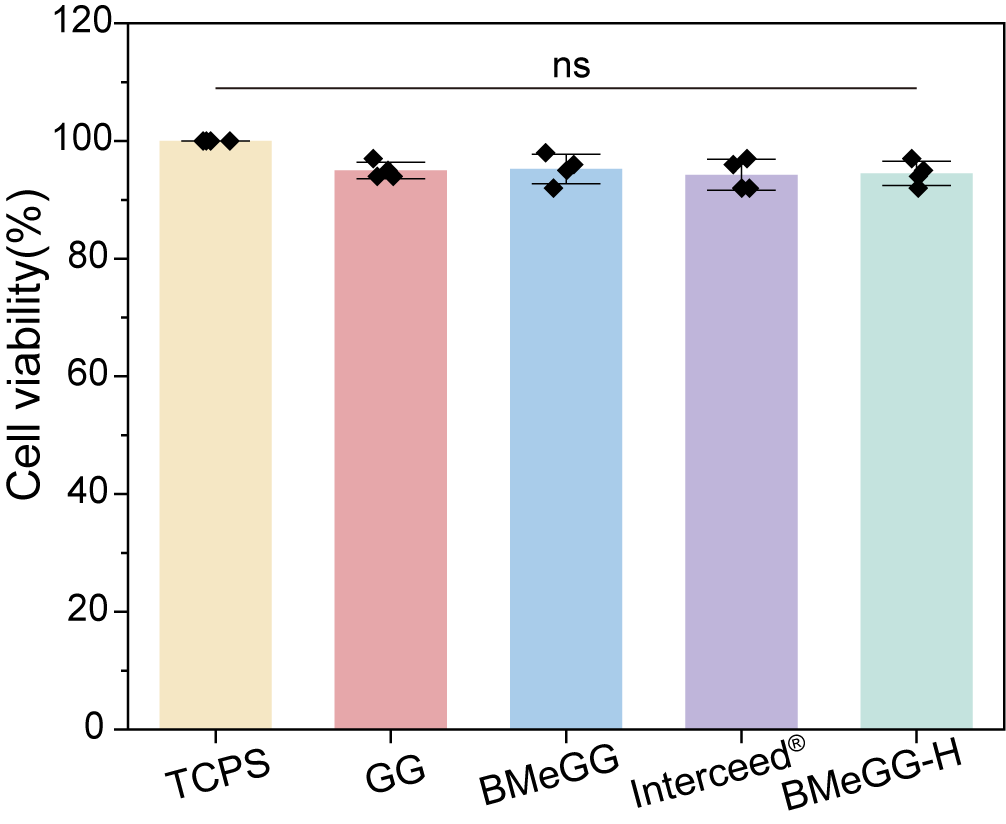


Figure S10. The cytotoxicity of BMeGG-H hydrogel was quantitatively analyzed using the Cell Counting Kit-8 (CCK-8). The cell viability of all groups was comparable to the tissue culture plate (TCP) group.

**
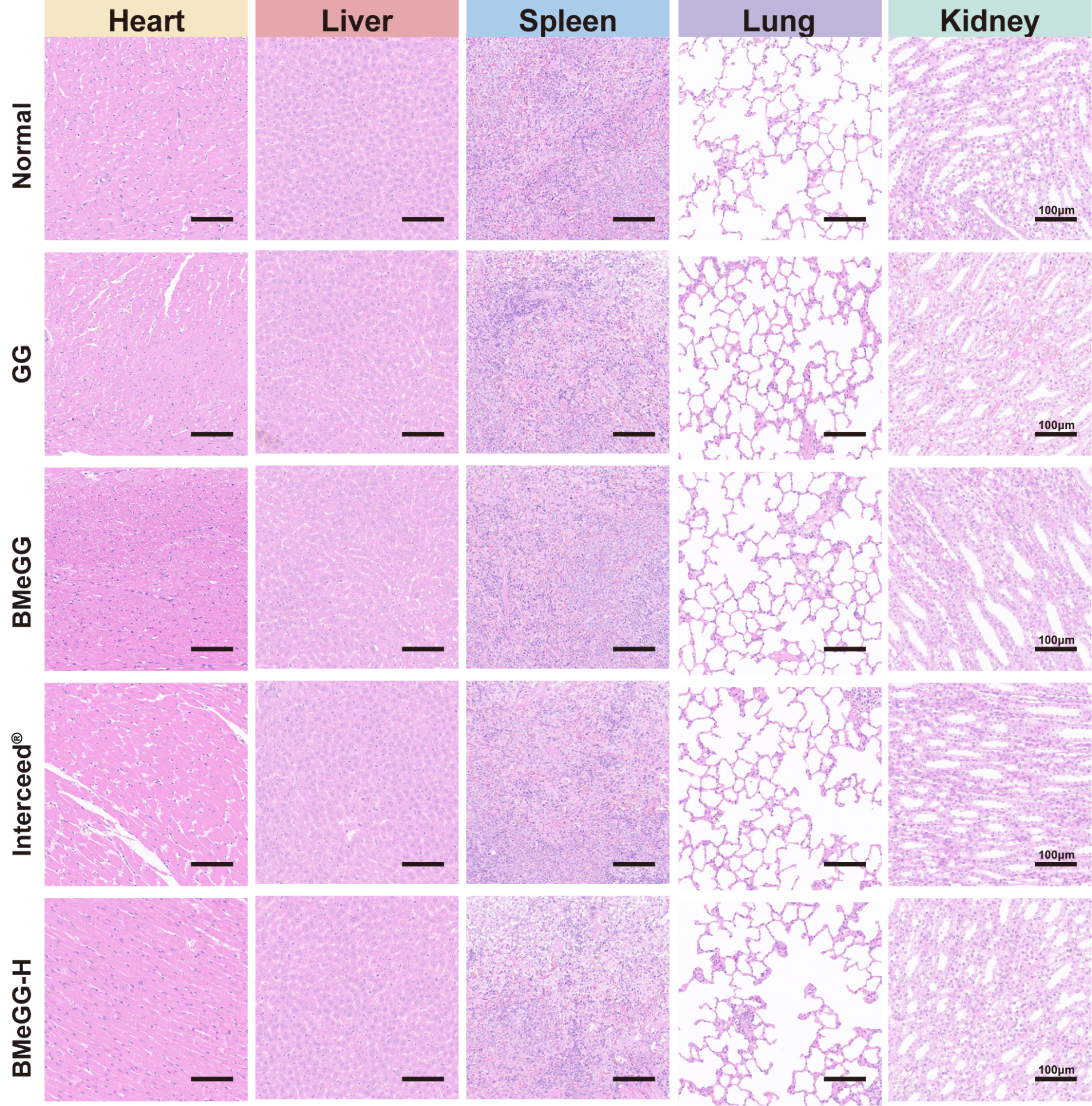
**

**Figure S11.** Representative images of H&E staining (heart, liver, spleen, lung, and kidney) demonstrated the *in vivo* biocompatibility of each gel 7 days post-implantation. Scale bars = 100 μm.

**
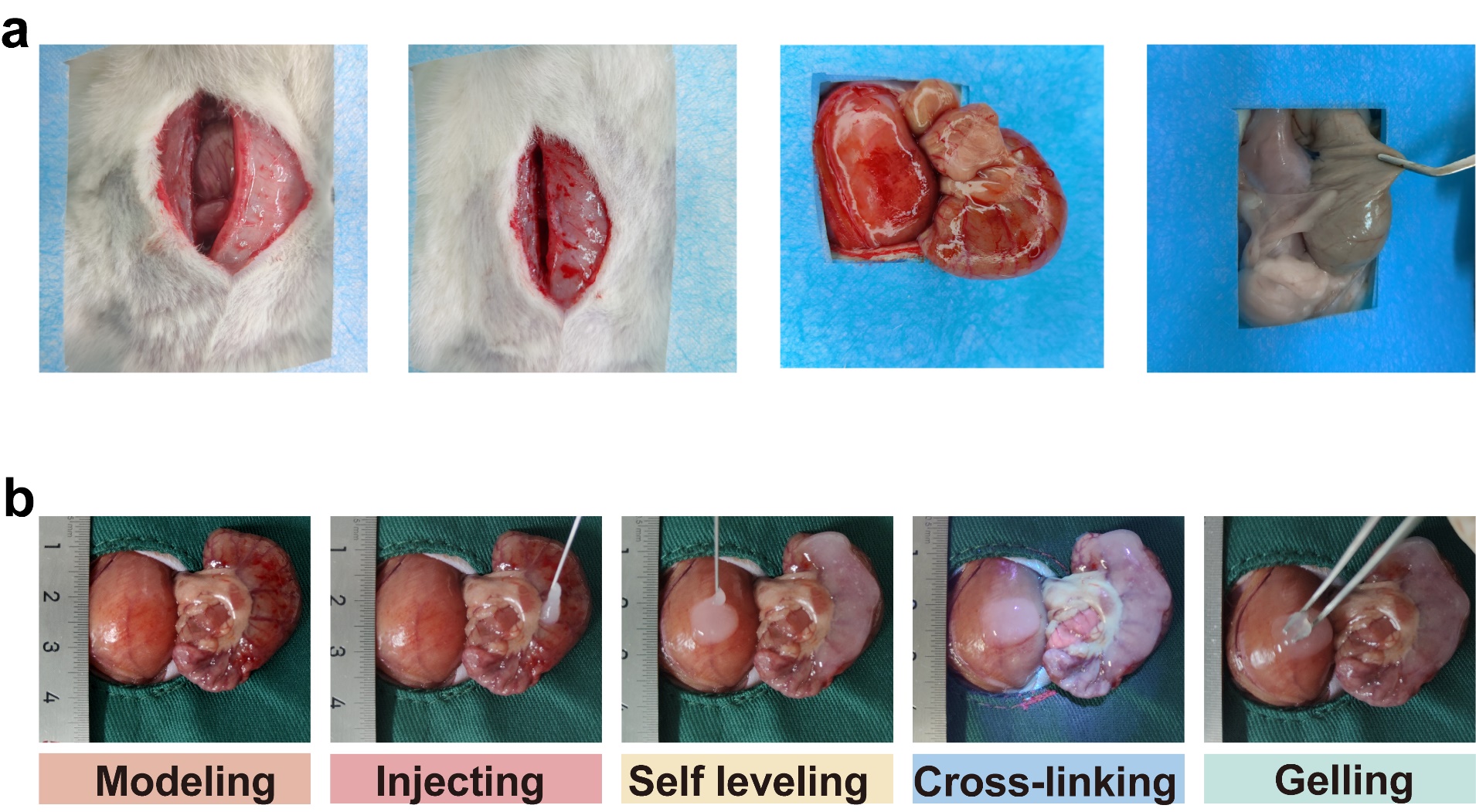
**

**Figure S12.** hematic diagram of the rat sidewall defect-cecum abrasion model.


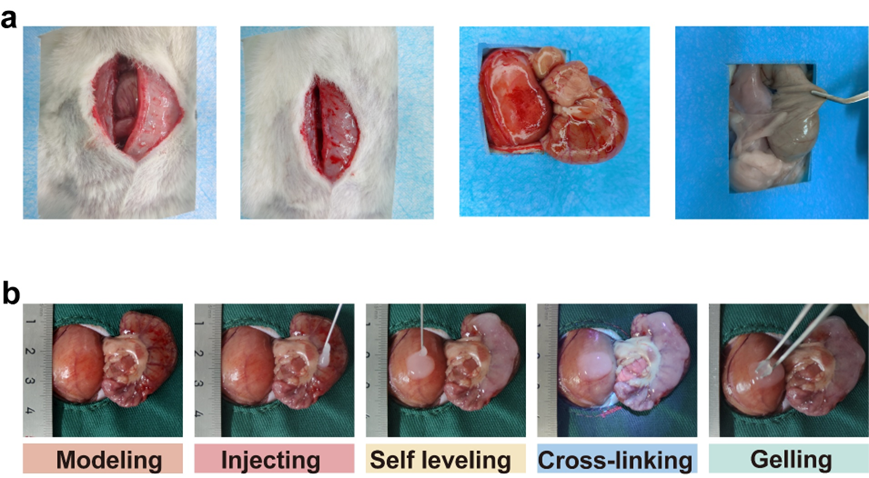


**Figure S13.** The application of BMeGG-H hydrogel in a cecal sidewall defect model.

**Table S4.** Double-blind scoring of tissue adhesions following a standard scoring system.

| Score | Events or phenomena |
| --- | --- |
| 0 | A smooth and intact surface without adhesion occurrence |
| 1 | A single-layer thin-film adhesion on the surface of the cecum |
| 2 | Multiple layers of thin-film adhesion present on the surface of the cecum |
| 3 | Localized adhesion on the cecum exceeding the thickness of the thin film |
| 4 | Multiple thick adhesions with localized adhesion attached to the surface of the cecum |
| 5 | The thick adhesion formed between the cecum and surrounding tissues has reached a vascularized degree |

**Table S5.** Peritoneal adhesion scores in rats 7 days after laparotomy.

| **Score** | Normal | Model | GG | BMeGG | | Interceed | BMeGG-H |
| --- | --- | --- | --- | --- | --- | --- | --- |
| 5 | 0 | 6 | 1 | | 0 | 0 | 0 |
| 4 | 0 | 2 | 2 | | 1 | 0 | 0 |
| 3 | 0 | 0 | 3 | | 1 | 0 | 0 |
| 2 | 0 | 0 | 1 | | 1 | 2 | 0 |
| 1 | 0 | 0 | 1 | | 2 | 2 | 1 |
| 0 | 8 | 0 | 0 | | 3 | 4 | 7 |

**Table S6.** Peritoneal adhesion scores in rats 14 days after laparotomy.

| Score | Normal | Model | GG | BMeGG | Interceed | BMeGG-H |
| --- | --- | --- | --- | --- | --- | --- |
| 5 | 0 | 7 | 2 | 1 | 0 | 0 |
| 4 | 0 | 1 | 3 | 1 | 1 | 0 |
| 3 | 0 | 0 | 2 | 2 | 1 | 0 |
| 2 | 0 | 0 | 1 | 1 | 1 | 1 |
| 1 | 0 | 0 | 0 | 1 | 1 | 1 |
| 0 | 8 | 0 | 0 | 2 | 4 | 6 |


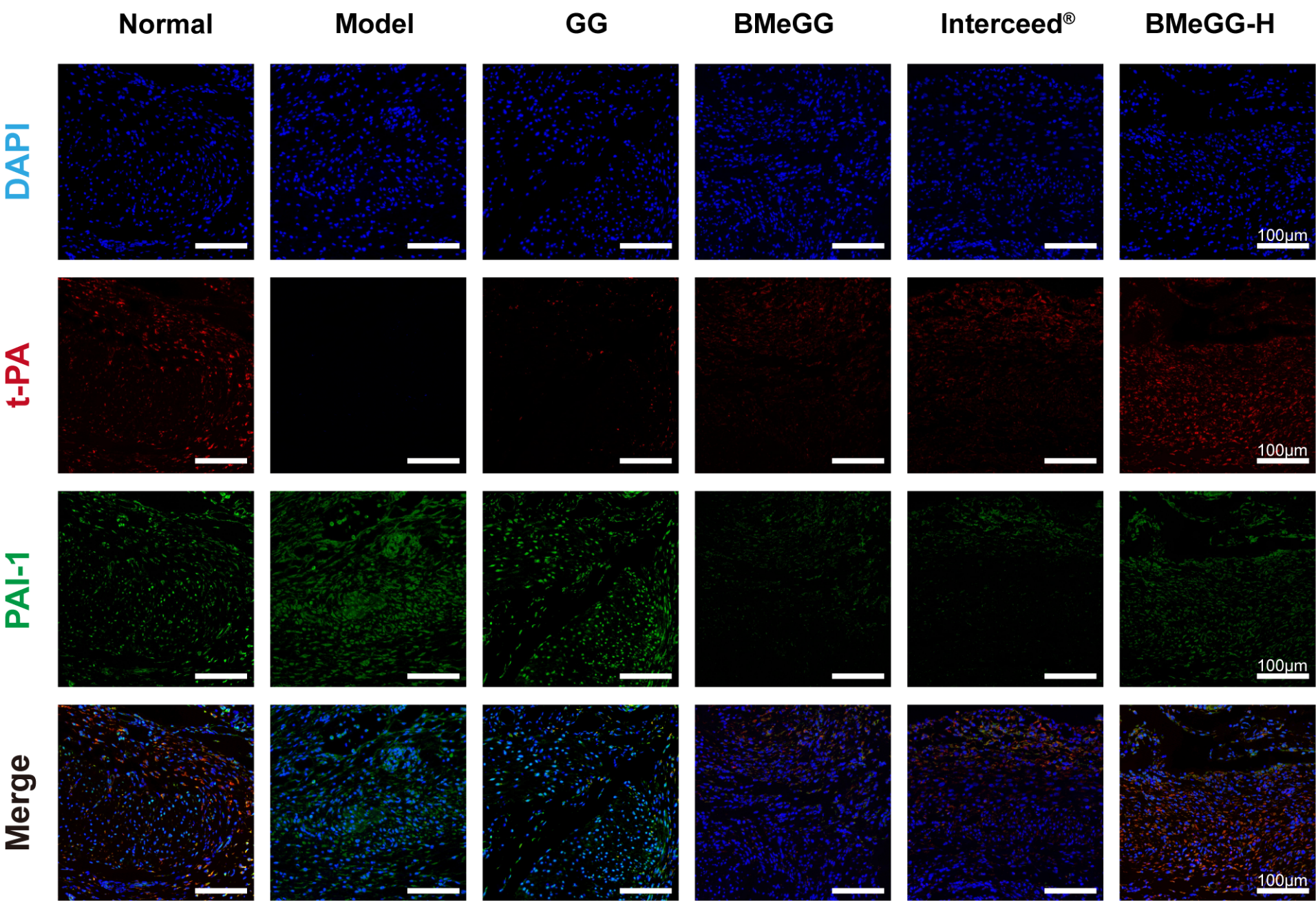


**Figure S14.** Immunofluorescence staining of DAPI (blue), t-PA-AlexaFluor594 (red) and PAI-1-AlexaFluor488 (green) in injured tissues on postoperative days 14.


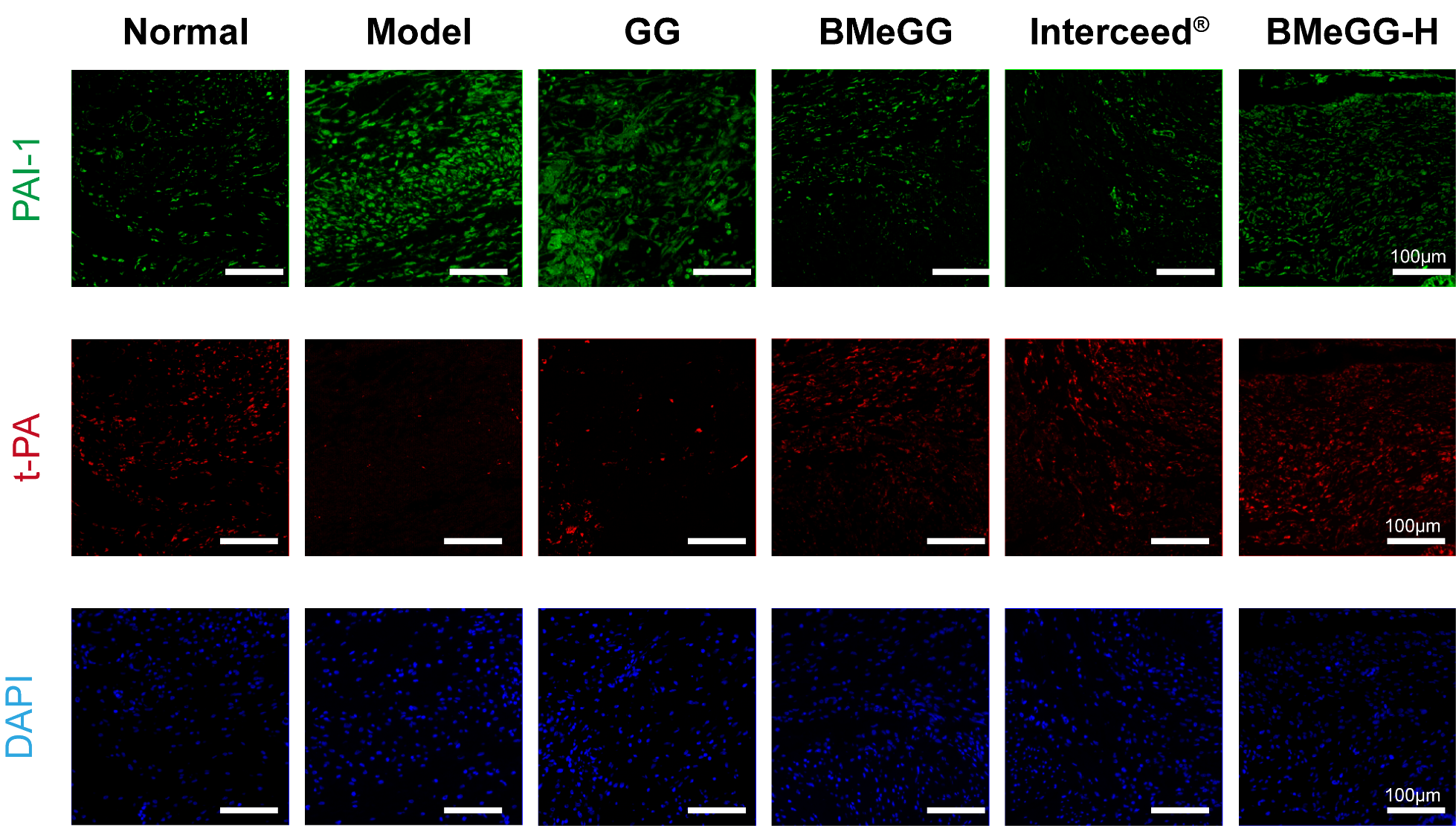


**Figure S15.** Single-channel image of immunofluorescence staining for DAPI (blue), t-PA-AlexaFluor594 (red) and PAI-1-AlexaFluor488 (green) in injured tissues on postoperative Day 7.


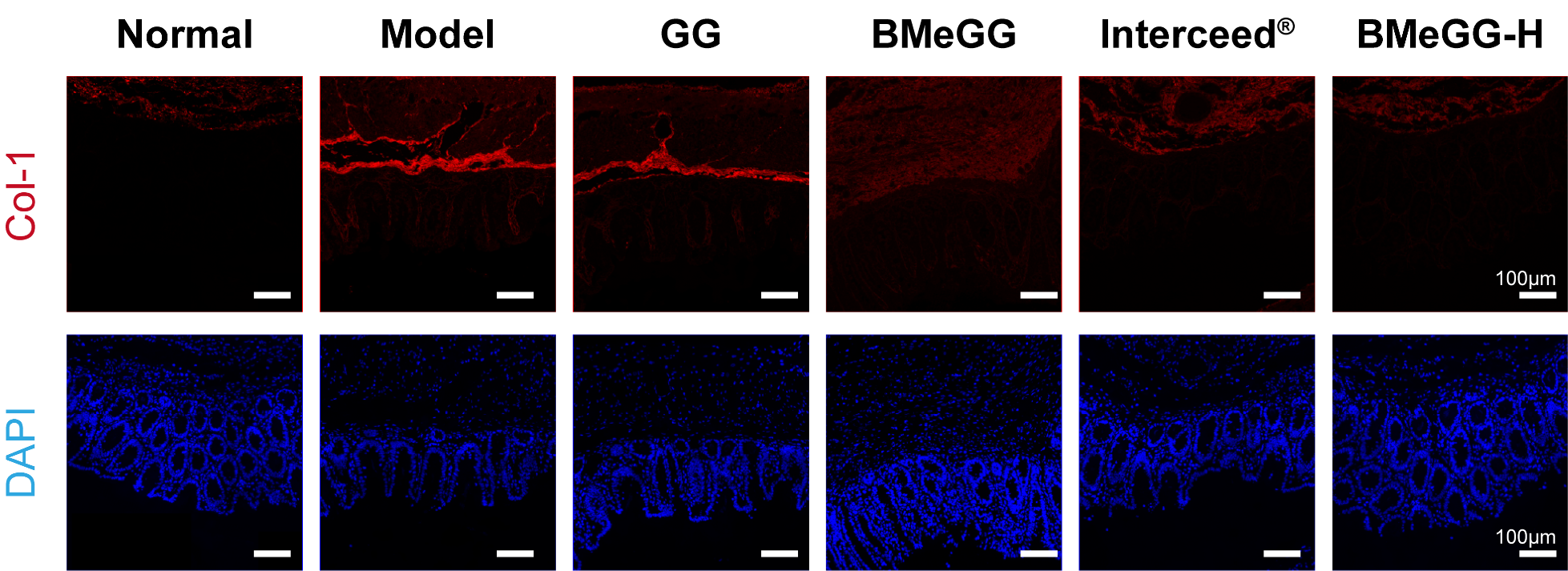


**Figure S16.** Single-channel image of immunofluorescence staining for DAPI (blue) and Col-1 (red) in injured tissue on postoperative Day 7.


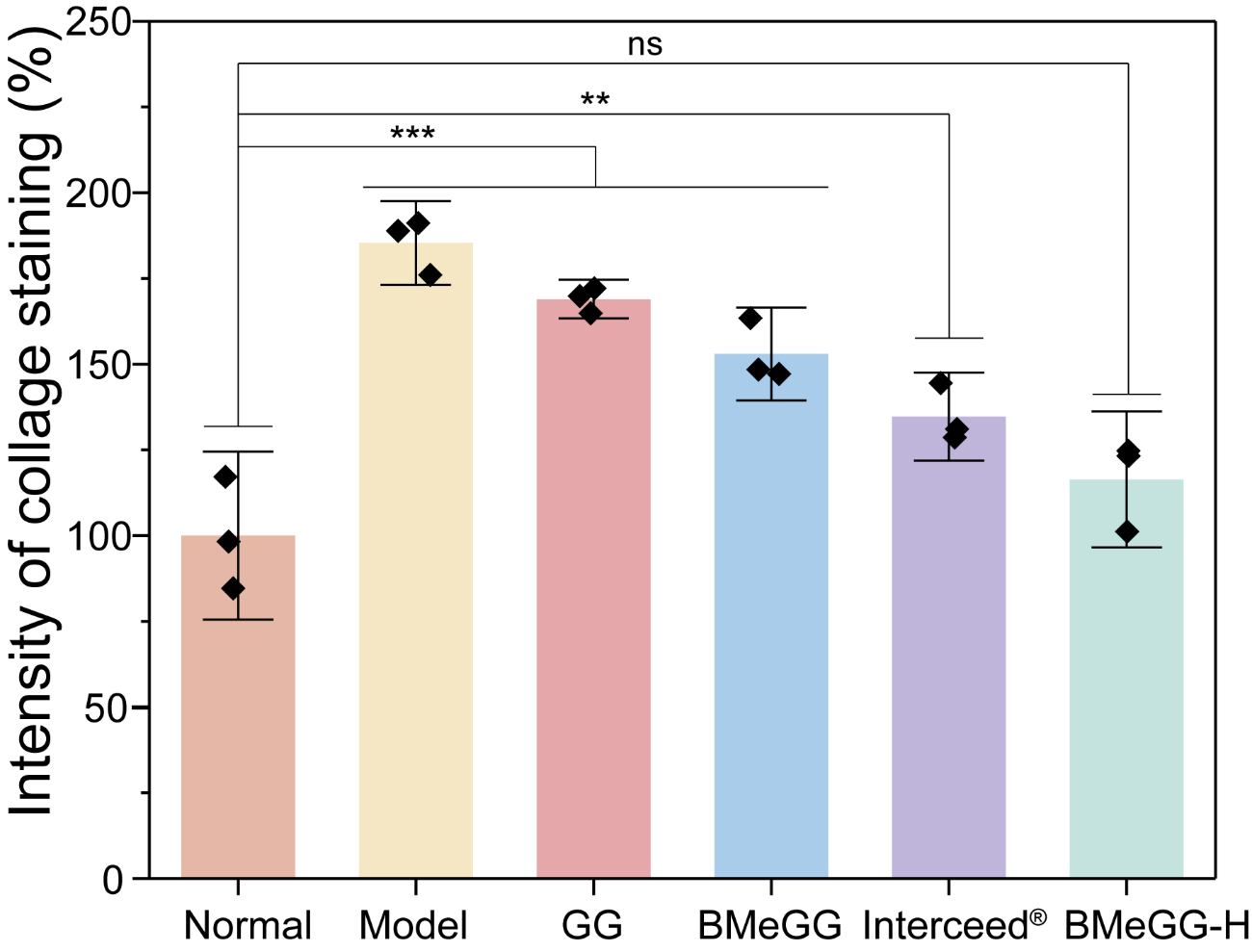


**Figure S17.** Collagen deposition statistics in injured rat cecum tissues on postoperative Day 7.


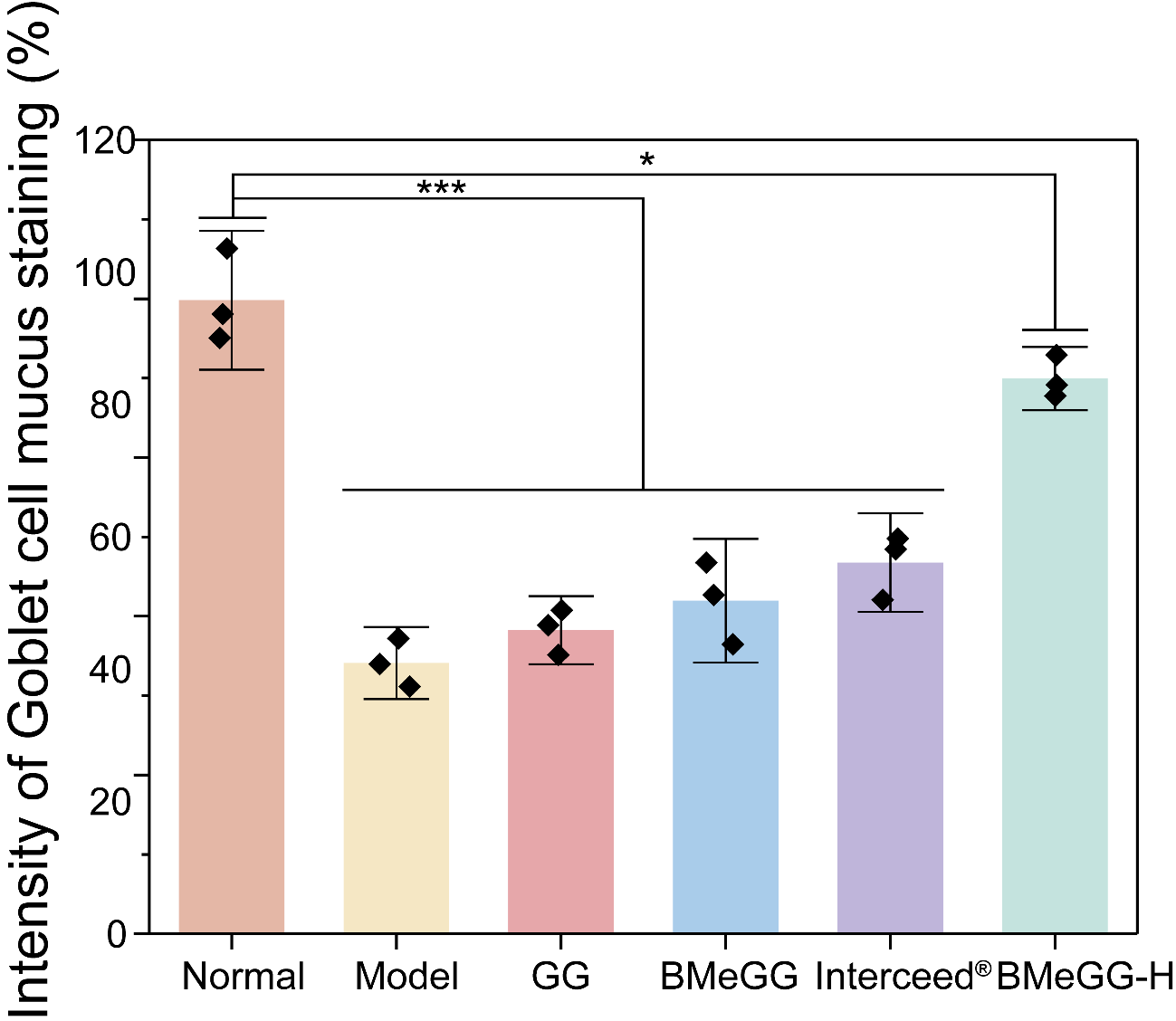


**Figure S18.** Relative mucin secretion from goblet cells in injured rat cecum tissues on postoperative Day 7.


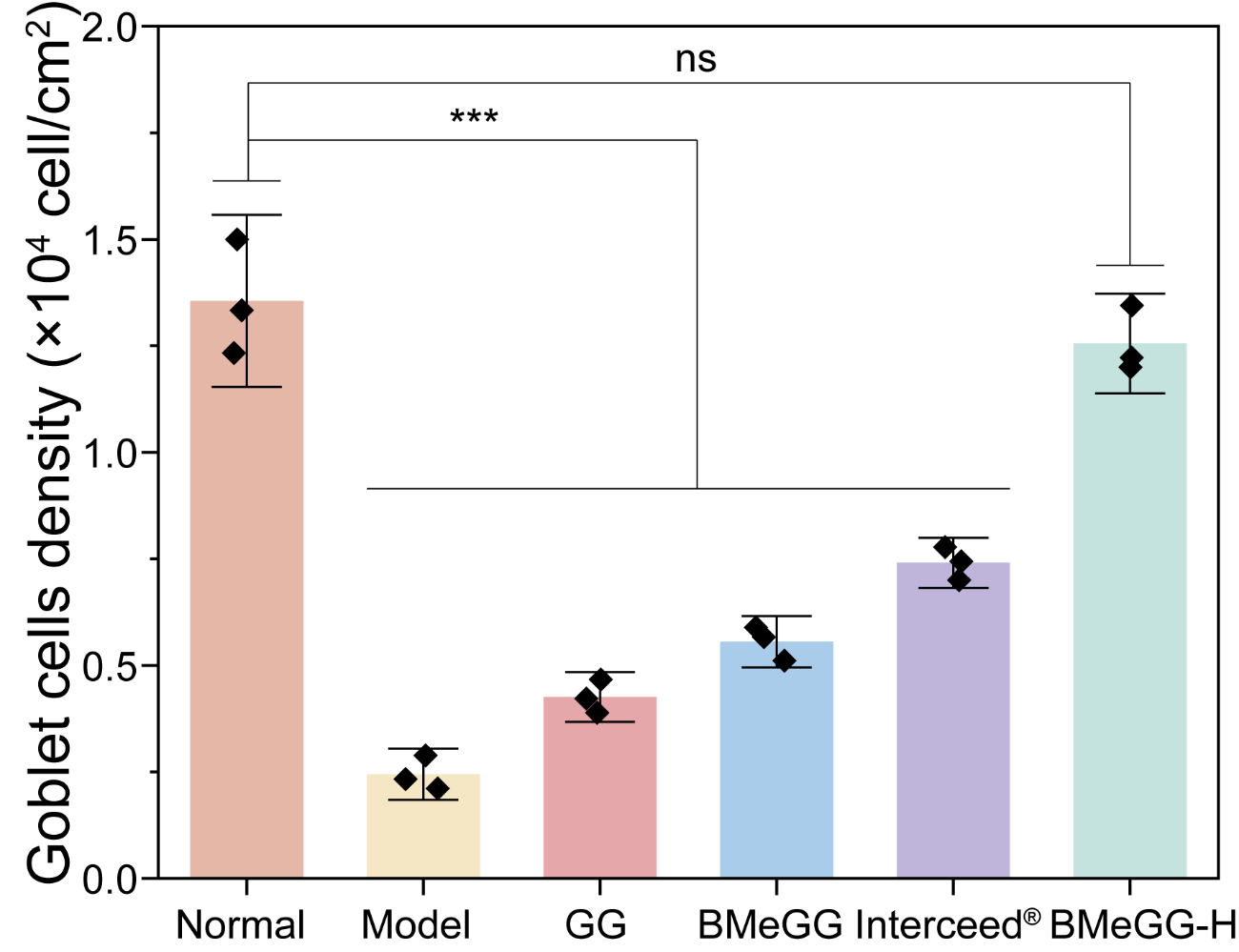


**Figure S19.** Quantification of goblet cells in injured rat cecum tissues on postoperative Day 7.

**Table** **S7.** Sequences of primers used in RT-qPCR

| Primer | Sequence (5’-3’) |
| --- | --- |
| TNF-α (forward primer) | GTCCCAACAAGGAGGAGAAGTT |
| TNF-α (reverse primer) | CTCCGCTTGGTGGTTTGCTA |
| TGF-β1 (forward primer) | CTGCTGACCCCCACTGATAC |
| TGF-β1 (reverse primer) | AGCCCTGTATTCCGTCTCCT |
| IL-6 (forward primer) | GCCCACCAGGAACGAAAGTC |
| IL-6 (reverse primer) | ACTGGCTGGAAGTCTCTTGCG |
| PAI-1 (forward primer) | CGGCACAATCCAACAGAGAC |
| PAI-1 (reverse primer) | ACACGTCCAGTTTTGTCCCA |
| t-PA (forward primer) | GGAAAGAAGCAAGCAAGGCAC |
| t-PA (reverse primer) | GAACCTCCTGTGTATTCCCTGG |
| ZO-1 (forward primer) | GCCAGCTTTAAGCCTCCAGA |
| ZO-1 (reverse primer) | TGGCTTCGCTTGAGGTTTCT |
| Claudin-1 (forward primer) | TGTGTCCACCATTGGCATGA |
| Claudin-1 (reverse primer) | ACTAATGTCGCCAGACCTGAAA |
| Occludin (forward primer) | GGGGCGCAGCAGGTCT |
| Occludin (reverse primer) | GCCTGTAAGGAGGTGGACTC |

# References

[1] J. T. Oliveira, L. Martins, R. Picciochi, P. Malafaya, R. Sousa, N. Neves, J. Mano, R. Reis, J. Biomed. Mater. Res., Part A 2010, 93, 852.

[2] M. Hamcerencu, J. Desbrieres, A. Khoukh, M. Popa, G. Riess, *Carbohydr. Polym.* **2008**, 71, 92.

[3] V. A. Basiuk, L. V. Henao-Holguín, *J. Comput. Theor. Nanosci.* **2014**, 11, 1609.

[4] H. Zhu, Y. Huang, J. Ren, B. Zhang, Y. Ke, A. K. Y. Jen, Q. Zhang, X. L. Wang, Q. Liu, *Adv. Sci.* **2021**, 8, 2003534.

[5] H. Wang, Y. Wu, C. Cui, J. Yang, W. Liu, *Adv. Sci.* **2018**, 5, 1800711.

[6] A. F. Shahraki, O. P. Yadav, H. Liao, *Int. J. Performability Eng.* **2017**, 13, 299.

[7] M. Manaargadoo-Catin, A. Ali-Cherif, J.-L. Pougnas, C. Perrin, *Adv. Colloid Interface Sci.* **2016**, 228, 1.

[8] W. Liang, W. He, R. Huang, Y. Tang, S. Li, B. Zheng, Y. Lin, Y. Lu, H. Wang, D. Wu, Adv. Mater. 2022, 34, 2108992.

[9] E. Zhang, J. Li, Y. Zhou, P. Che, B. Ren, Z. Qin, L. Ma, J. Cui, H. Sun, F. Yao, Acta Biomater. 2017, 55, 420.

[10] C. Seillier, P. Hélie, G. Petit, D. Vivien, D. Clemente, B. Le Mauff, F. Docagne, O. Toutirais, *Cell. Immunol.* **2022**, 371, 104451.
